# Nuclear receptors in ovarian cancer: changing paradigms in cancer therapeutics

**DOI:** 10.3389/fonc.2024.1383939

**Published:** 2024-07-15

**Authors:** Anjana Sajeev, Bandari BharathwajChetty, Mukesh Kumar Manickasamy, Mohammed S. Alqahtani, Mohamed Abbas, Mehdi Shakibaei, Gautam Sethi, Zhaowu Ma, Ajaikumar B. Kunnumakkara

**Affiliations:** ^1^ Cancer Biology Laboratory, Department of Biosciences and Bioengineering, Indian Institute of Technology Guwahati (IITG), Guwahati, Assam, India; ^2^ Radiological Sciences Department, College of Applied Medical Sciences, King Khalid University, Abha, Saudi Arabia; ^3^ BioImaging Unit, Space Research Centre, Michael Atiyah Building, University of Leicester, Leicester, United Kingdom; ^4^ Electrical Engineering Department, College of Engineering, King Khalid University, Abha, Saudi Arabia; ^5^ Chair of Vegetative Anatomy, Ludwig-Maximilians-University Munich, Munich, Germany; ^6^ Department of Pharmacology, Yong Loo Lin School of Medicine, National University of Singapore, Singapore, Singapore; ^7^ National University of Singapore (NUS) Center for Cancer Research, Yong Loo Lin School of Medicine, National University of Singapore, Singapore, Singapore; ^8^ School of Basic Medicine, Health Science Center, Yangtze University, Jingzhou, Hubei, China

**Keywords:** ovarian cancer, nuclear receptors, targeted therapies, clinical trials, chemoresistance

## Abstract

Ovarian cancer (OVC) is one of the most common causes of cancer-related deaths in women worldwide. Despite advancements in detection and therapy, the prognosis of OVC remains poor due to late diagnosis and the lack of effective therapeutic options at advanced stages. Therefore, a better understanding of the biology underlying OVC is essential for the development of effective strategies for early detection and targeted therapies. Nuclear receptors (NRs) are a superfamily of 48 transcription factors that, upon binding to their specific ligand, play a vital role in regulating various cellular processes such as growth, development, metabolism, and homeostasis. Accumulating evidence from several studies has shown that their aberrant expression is associated with multiple human diseases. Numerous NRs have shown significant effects in the development of various cancers, including OVC. This review summarizes the recent findings on the role of NRs in OVC, as well as their potential as prognostic and therapeutic markers. Further, the basic structure and signaling mechanism of NRs have also been discussed briefly. Moreover, this review highlights their cellular and molecular mechanisms in chemoresistance and chemosensitization. Further, the clinical trials targeting NRs for the treatment of OVC have also been discussed.

## Graphical Abstract

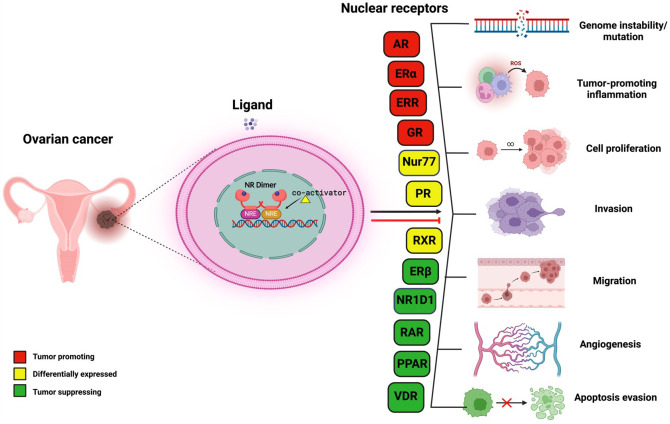

## 1 Introduction

Ovarian cancer (OVC) is one of the most intractable diseases, with an increasing number of mortalities in women worldwide (1, 2). As per the report of GLOBOCAN 2020, OVC ranks eighth in terms of both the number of new cases (313,959) and deaths (207,252) across the world (3). Even though chemotherapy and surgery have proven effective against OVC over the past few years, their undesirable side effects that affect the quality of life of patients including fatigue, neurotoxicity, and tumor recurrence, pose a major concern (4–6). Despite the emergence of various multimodal treatment strategies such as immunotherapy and targeted therapies, OVC remains life-threatening due to its high molecular heterogeneity, peritoneal dissemination, and late-stage diagnosis (2, 7, 8). Moreover, the chemoresistance of tumor cells also stands as the Achilles’ heel in overcoming the consequences of this disease (9). Hence, there is an imperative need for the identification of potential targets and novel drugs that could lead to the development of safe, efficacious, and innovative therapeutic strategies circumventing OVC. As nuclear receptors (NRs) play a pivotal role in the development of different malignancies, in the present review, we have highlighted the role of NRs in the development and progression of OVC and the potential of agonists and antagonists of NRs for the treatment of this cancer. In addition, the compounds derived from natural sources also showed potential effects in the prevention and treatment of different cancers, including OVC (10–14). Hence, we have also included the role of natural products in modulating NRs in OVC cells.

**Figure 1:**
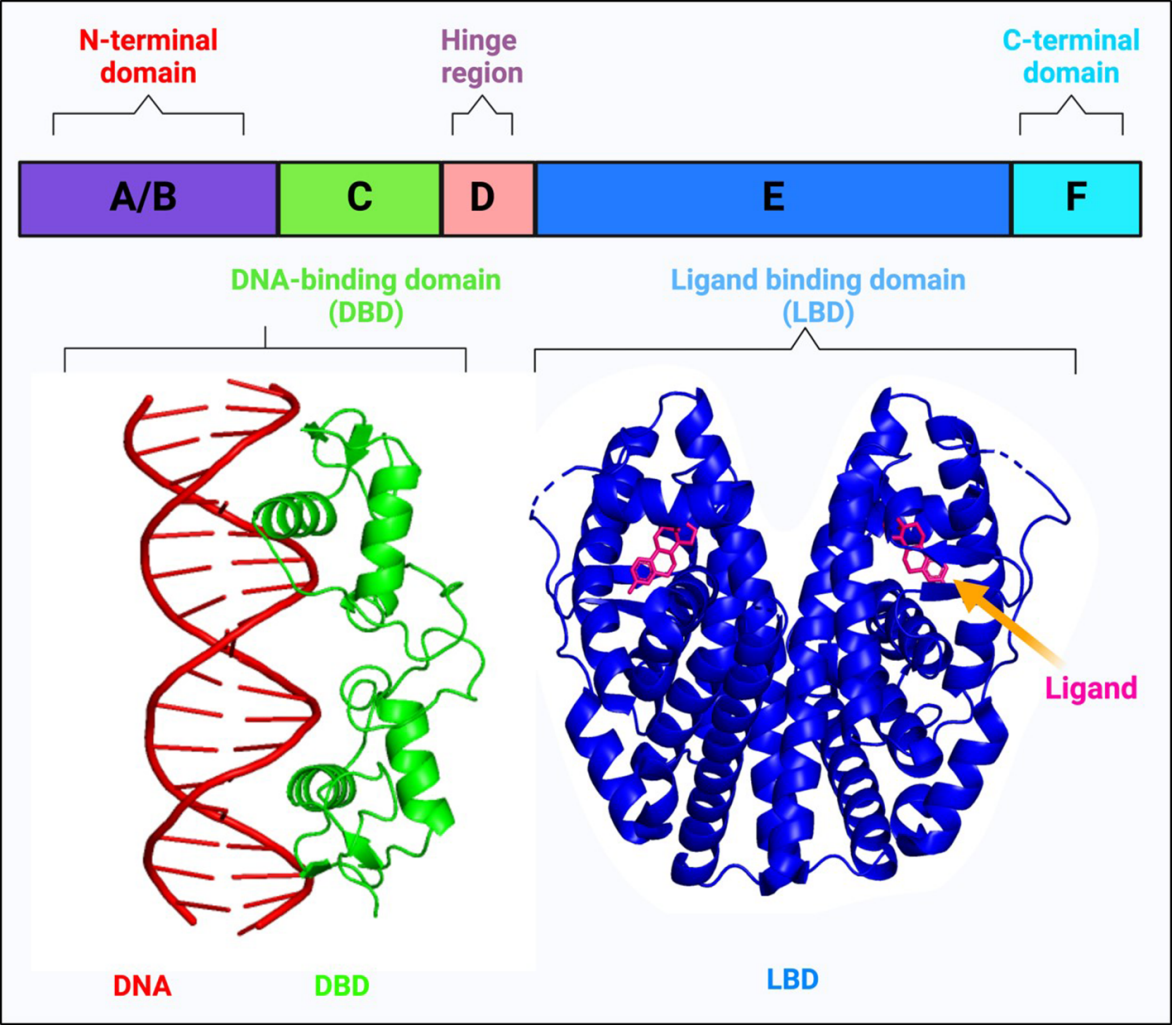
Schematic representation of the basic structure of a nuclear receptor, highlighting its different domains: The N-terminal domain **(A, B)**, the DNA-binding domain **(C)**, the hinge region **(D)**, the ligand-binding domain **(E)**, and the C-terminal domain **(F)**. The DNA-binding domain (DBD) interacts with a double-stranded DNA helix shown in red (The crystal structure of the estrogen receptor DNA-binding domain bound to DNA, PDB ID: 1HCQ). This interaction is critical for the nuclear receptor’s ability to regulate gene expression. The structure of the ligand-binding domain (LBD) demonstrates how ligands can bind to nuclear receptors, influencing their conformation and function (Structure of complex between human estrogen receptor alpha-LBD in complex with 17-β-estradiol, PDB ID: 1GWR is depicted). The ligands typically modulate the receptor’s activity, affecting its ability to interact with co-regulators and its gene-regulating capability (25–29).

**Figure 2:**
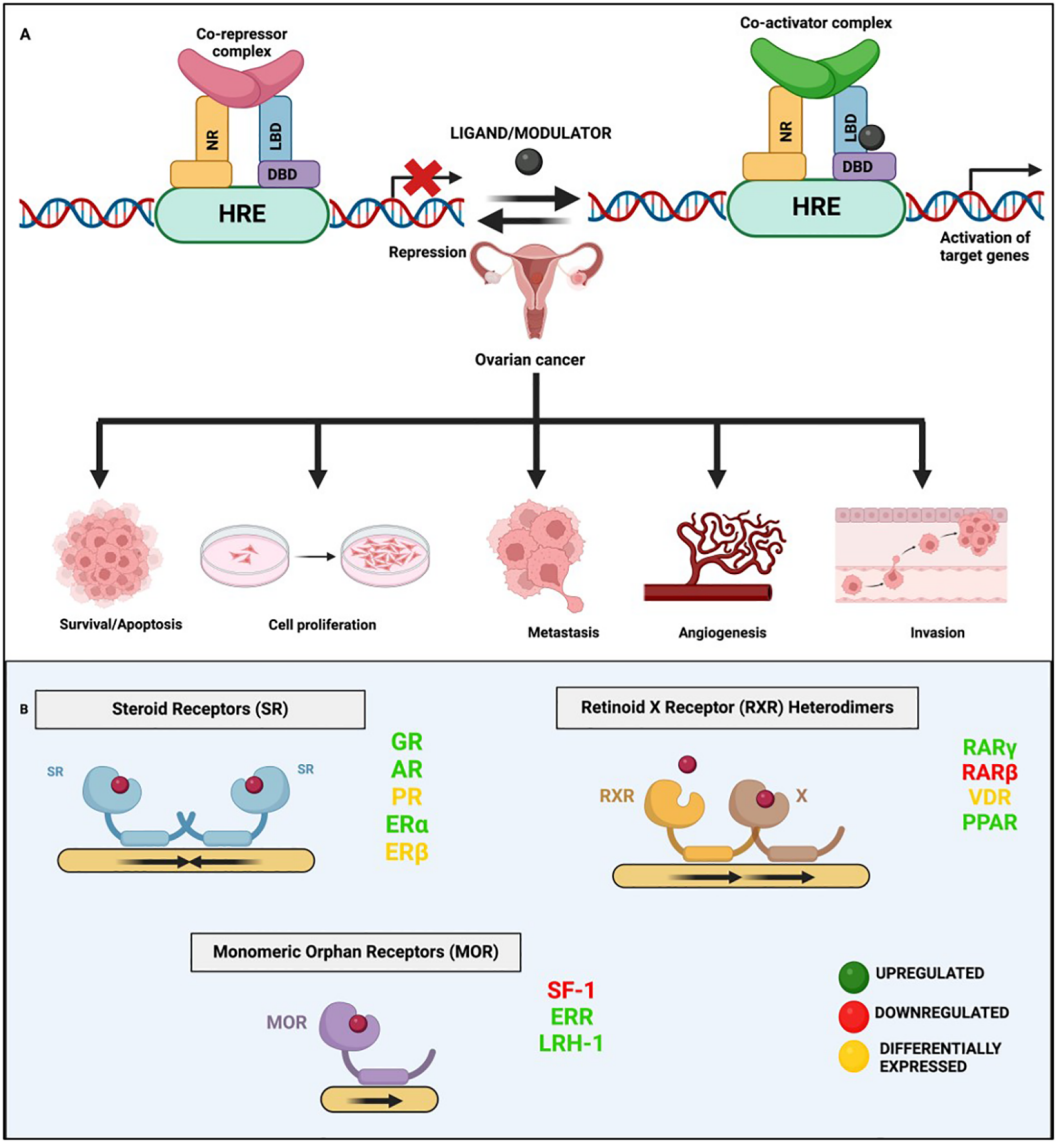
General mechanism of nuclear receptor signaling. **(A)** This panel illustrates the dual roles of NRs in gene regulation via binding to hormone response elements (HREs). In the case of Type II NR signaling, in the absence of a ligand/modulator, the NR forms a complex with co-repressors that bind to HREs, repressing gene expression. Upon ligand binding, the receptor undergoes a conformational change, displacing co-repressors and recruiting co-activators, leading to the activation/suppression of target genes including the ones involved in various hallmarks of OVC. **(B)** This panel depicts the different types of NRs implicated in OVC and the general mechanism of their binding as monomers, homo- or hetero- dimers.

NRs are members of a large superfamily of transcription factors (TFs) that are present in all metazoans, except plants and yeast (15, 16). These proteins are thought to have a significant role in maintaining homeostasis, immunoregulation, and regular physiological processes like cell growth and differentiation (15, 17–21). Most of these receptors have well-defined ligands, apart from a few, named ‘orphan receptors’ which lack specific ligands. Unlike other TFs, NR’s activity can be regulated according to the ligand that binds to it. Their ligands are small lipophilic molecules that include thyroid hormone, some oxysterols, retinoic acid (RA), or steroid hormones like estrogen and progesterone that could permeate through the cell membrane and interact with NRs in the cytoplasm (22, 23). NRs, upon activation, modulate the transcription of numerous genes that are involved in essential biological activities such as cell differentiation, circadian functions, metabolism, reproduction, etc (24). NRs perform integrative roles in regulating the transcription of different genes in various cell types and tissues that control development and homeostasis (15, 23). The basic structure of NRs includes a variable A/B domain at the amino-terminal, which contains an activation function 1 (AF1) that interacts with various co-regulator proteins, a C domain which is a centrally conserved DNA-binding domain (DBD) with two zinc finger motifs, a D domain that serves as a short hinge region between DBD and ligand binding domain (LBD) which also facilitates nuclear localization and a fairly well-conserved E domain, found at the carboxy-terminal (summarized in **Figure 1**) (30). Ligands, along with their coactivators or corepressors control the action of NRs by regulating the activation function 2 (AF2) present at the LBD (31). Selective modulation of AF2 helix by specific synthetic agonists/antagonists can alter the activities of NRs in a favorable manner. For example, it was observed that the binding of estrogen antagonist, dihydroxytamoxifen results in a distorted configuration of AF2 helix (**Figure 2A**) (31).

Clear lines of evidence have emerged elucidating the role of NRs in various pathologies, including asthma, diabetes, rheumatoid arthritis, and different cancers, including OVC (32–36). Multiple studies have proved the role of NRs in regulating significant hallmarks of cancer, such as proliferation, survival, epithelial-mesenchymal transition (EMT), invasion, migration, apoptosis, etc (37–44). NRs exhibit differential expression in cancers, where some act as oncogenes while others act as tumor suppressors (45–47). Numerous NRs like estrogen receptor (ER), peroxisome-proliferator-activator receptor (PPAR), progesterone receptors (PR), retinoic acid receptors (RAR), and vitamin D receptors (VDR) have been linked to several malignancies (48–51). Many preclinical and clinical studies have revealed the role of dysregulated NRs as therapeutic targets for OVC. Consequently, there is an opportunity to develop selective agonists and antagonists for these receptors (22, 23, 31). Over recent years, there has been a quantum leap in the development of several drugs for the treatment of OVC that specifically modulate NR signaling in tumor cells. For instance, letrozole, an aromatase (an enzyme that converts androgens to estrogens) inhibitor, is undergoing Phase III clinical trial for the treatment of OVC due to the high level of estrogen (52). Although several studies have uncovered the role of NRs as important molecular markers and targets in diagnosis and therapy, there is currently no comprehensive compilation of the various preclinical and clinical studies on NRs in OVC. Therefore, in this review, we focus on the role of NRs in the development of OVC, the modulation of NRs by multiple agonists or antagonists, their mechanistic underpinnings, and the ongoing clinical trials delineating their potential as a therapeutic option for clinical management of this deadly cancer.

## 2 NRs in OVC

**Figure 3:**
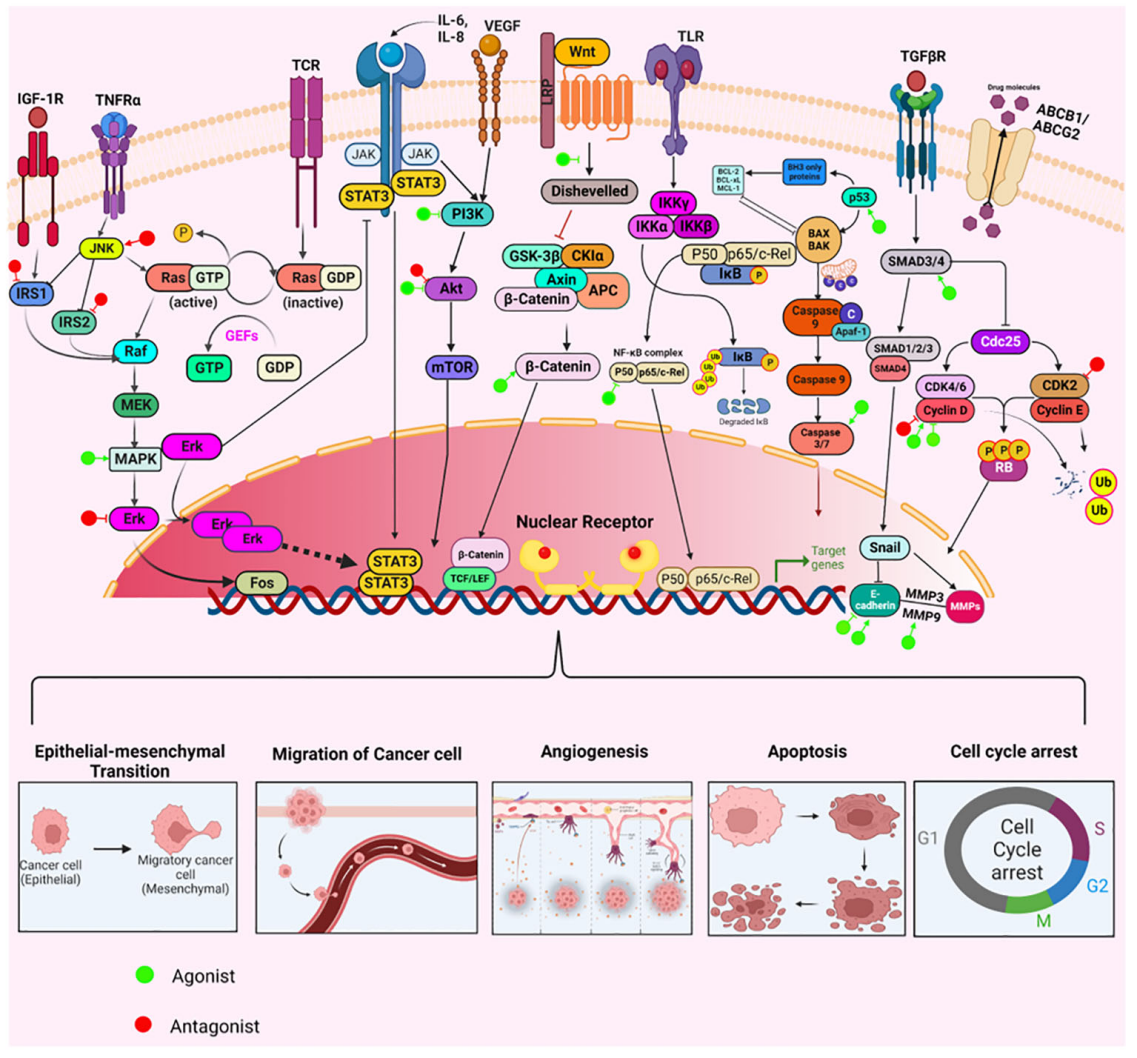
The mechanisms through which agonists or antagonists of various nuclear receptors influence different cellular processes regulating ovarian cancer pathogenesis. These compounds modulate key cellular behaviors like survival, proliferation, epithelial-mesenchymal transition (EMT), invasion, angiogenesis, migration, apoptosis, and cell cycle arrest. This modulation occurs through the upregulation or downregulation of specific pathways integral to the development and progression of ovarian cancer.

NRs have long been at the cutting edge of cancer research, where they are known to play crucial roles in regulating these malignancies, including OVC. The fact that many of the identified NRs are responsive to hormones and can bind with drugs or small molecules makes them potential targets for OVC therapeutics. NRs regulate differentiation and development and maintain homeostasis in normal cells, but they control cellular growth, apoptosis, invasion, and migration, by dysregulating major signaling processes in a variety of OVC cell types. For instance, few of the NRs, primarily hormone/steroid receptors including ER and androgen receptor (AR), show tremendous upregulation, but others, such as VDR and RAR, are downregulated or differentially expressed in OVC (**Figure 2B**) (53–56). The expression of various NRs in OVC is depicted in **Table 1** (49, 53–55, 57–74). Hence, we attempt to provide a deeper comprehension of the multifaceted aspects of variable expression of NRs in OVC tumor cells and their underlying molecular mechanism upon agonist/antagonist binding, (**Table 2**) which envisages their significant role in the treatment of OVC. The mechanism of action of NRs upon agonist/antagonist binding is briefly depicted in **Figure 3**.

**Table 1:** Nuclear receptor (NR) expression in ovarian cancer.

*In vitro/In vivo*/Clinical	Cell lines/Models/Tissues	Expression (Up/Down-regulation)	Reference
Androgen Receptor (AR)			
Clinical	Ovarian teratocarcinoma tissues	Up	(57)
Clinical	Epithelial ovarian cancer tissues	Up	(54)
Estrogen Receptor (ER)			
Clinical	Ovarian tumor tissues	Increased activity	(58)
Clinical	Ovarian epithelial cancer tissues	Up	(59)
ERα			
Clinical	BRCA-associated ovarian carcinoma tissues	Up	(53)
Clinical	Ovarian serous cystadenocarcinoma tissues	Up	(60)
Clinical	Human benign and malignant ovarian cancer tissues	Increase in ERα:ERβ mRNA ratio	(61)
Clinical	Primary and metastatic epithelial ovarian cancer tissues	Up	(55)
Clinical	Ovarian tumor tissues	Up	(62)
ERβ			
Clinical	Ovarian serous cystadenocarcinoma tissues	Down	(60)
Clinical	Human benign and malignant ovarian cancer tissues	Increase in ERα:ERβ mRNA ratio	(61)
Clinical	Primary and metastatic epithelial ovarian cancer tissues	Down	(55)
Clinical	Ovarian tumor tissues	Down	(62)
Estrogen Related Receptor (ERR)			
Clinical	Serous ovarian tumor tissues	Up	(63)
Clinical	Ovarian cancer patient tissues	Up	(64)
Glucocorticoid Receptor (GR)			
Clinical	Ovarian tumor tissues	Increased activity	(58)
Liver Receptor Homolog-1 (LRH-1)			
Clinical	Ovarian granulosa cell tumor tissues	Up	(65)
Peroxisome Proliferator-Activated Receptor (PPAR)			
Clinical	Epithelial ovarian carcinoma tissues	Up	(66)
Clinical	Epithelial ovarian tumor tissues	Up	(67)
Progesterone Receptor (PR)			
Clinical	Malignant epithelial neoplasm tissues	Down	(59)
PR-A			
*In vitro*	OVCA 429, OVCA 432	Up	(68)
PR-B			
*In vitro*	OVCA 429, OVCA 432	Up	(68)
Retinoic Acid Receptor (RARβ)			
Clinical	Epithelial ovarian carcinoma tissues	Down	(69)
RARγ			
Clinical	Ovarian cancer tissues	Up	(49)
Steroidogenic Factor-1 (SF-1)			
*In vitro*	SKOV3, BG-1 and CAOV-3	Down (when compared with normal cells)	(70)
Vitamin D Receptor (VDR)			
Clinical	Ovarian carcinoma tissues	Up	(71)
Clinical	Epithelial ovarian carcinoma tissues	Up	(72)
Clinical	Primary ovarian carcinoma tissues	Down	(73)
Clinical	Primary ovarian carcinoma tissues	Down	(74)

**Table 2:** Mechanistic role of various nuclear receptors in ovarian cancer in the presence of their agonists/antagonists.

*In vitro/In vivo/Ex vivo*	Cell lines/Models	Intervention	Mode of action/Outcomes	References
Androgen Receptor				
*In vitro*	OVC NOVA, OV1225 cells	Flutamide	↓Cell proliferation	(75)
*In vitro*	OVC NOVA, OV 1225, OV 166 cells	DHT+ Epostane	↑AR ↓Cell proliferation	(75)
*In vitro*	OV 166 cells	DHT+ Flutamide/OH-Flutamide/Anandron	↑AR, Sensitivity ↓Cell survival	(75)
*In vitro*	OV 166 cells (Sensitive to anti-androgens)	Flutamide	↓Sensitivity	(75)
*In vivo*	BALB/c nude mice (OVA-5) xenograft	5-α-DHT	↑Tumor growth	(76)
*In vitro*	OVCAR-3 cells	1,25(OH)2D3	↑AR ↓Cell growth	(77)
*In vitro*	SKOV3 cells	DHT	↑ Cell proliferation, IL-6, IL-8	(78)
*In vitro*	SKOV3 cells	DHT+ Flutamide	↓ Cell proliferation, IL-6, IL-8	(78)
*In vitro*	OvCa 429 cells	MPA	↓MMP-2&-9	(79)
*In vitro*	OvCa cells (AR OE)	DHT	↑AR, Nuclear localization of AR ↓MMP-2&-9	(79)
*In vitro*	OvCa cells (AR OE)	MPA	↑AR, Nuclear localization of AR, Invasion of cells ↓MMP-2&-9	(79)
*In vitro*	OVCAR-3 cells	DHT	↑AR, PARP, Cell proliferation, Cells in S-phase, GNA13, ELKS, GSTP1, RERG, Rab25, Rab45, Rab35	(80)
*In vitro*	OVCAR-3, OSEC2 cells	Casodex	↓AR, Cell proliferation	(80)
*In vitro*	OVCAR-3 cells	DHT+ Casodex	↑AR	(81)
*In vitro*	OVCAR-3 cells	DHT+ siSGTA	↑AR (in nucleus) ↓SGTA	(81)
*In vitro*	PA-1 cells	AR cDNA	↑AR, Cell growth, CD133^+^ cells, Sphere number and size	(57)
*In vitro*	PA-1 cells	siAR	↓AR, Cell growth rate, CD133^+^ cells, Sphere number and size	(57)
*In vitro*	PA-1 cells (CD133^+^)	–	↑AR, CD133, CD24, OCT-4, Nanog, CD117, CD44	(57)
*In vitro*	PA1 cells	AR cDNA+ verapamil	↑Side population	(57)
*In vitro*	PA1 cells	siAR+ verapamil	↓Side population	(57)
*In vitro*	PA1 cells (CD133^+^)	ARcDNA	↑Sphere formation ↓p53, p16	(57)
*In vitro*	PA1 cells (CD133^+^)	siAR	↓Sphere formation	(57)
*In vitro*	SKOV3 cells (Taxol-resistant)	Taxol	↑AR, FKBP5, Nuclear translocation of AR	(82)
*In vitro*	SKOV3 cells (Taxol-resistant)	DHT	↓AR-FKBP5 interaction	(82)
*In vitro*	SKOV3 cells (Taxol-resistant)	shAR/shFKBP5	↑Taxol sensitivity ↓Cell viability	(82)
*In vitro*	SKOV3 cells (Taxol-resistant)	shAR	↓Cell viability, ABCB1, BMP5, FAT3, FGFR2, H1F0, SRCRB4D, STAG3, TMPRSS15	(82)
*In vitro*	SKOV3 cells (Taxol-resistant)	R1881	↑ABCB1, BMP5, FAT3, FGFR2, H1F0, SRCRB4D, STAG3, TMPRSS15	(82)
*In vitro*	SKOV3 cells (Taxol-resistant)	Taxol	↑AR, Cell viability, p-JNK ↓p-Akt, p-p38, cleaved Caspase-3, cleaved PARP	(83)
*In vitro*	SKOV3 cells (Taxol-resistant)	shAR	↑Apoptosis, JNK, Caspase-3, cleaved PARP ↓AR mRNA expression, Cell viability, ABCB1, TMPRSS15, FGFR2, H1F0, FAT3, BMP5, SRCRB4D, ABCG2, ABCB6	(83)
*In vitro*	SKOV3 cells (Taxol-resistant)	DHT	↑AR, ABCB1, TMPRSS15, FGFR2, HIF0, FAT3, BMP5, SRCRB4D, ABCG2, ABCB6	(83)
*In vitro*	SKOV3 cells (Taxol-resistant)	Paclitaxel	↑AR, ABCB1, H3K9Ac, H3KI4Ac, H3Ac, p300	(84)
*In vitro*	MDAH2774, TOV21G cells	Paclitaxel	↑AR, ABCB1	(84)
*In vitro*	SKOV3 cells (Taxol-resistant)	Paclitaxel+ shAR	↑Paclitaxel sensitivity ↓Cell viability	(84)
*In vitro*	SKOV3 cells (Taxol-resistant)	DHT	↑ABCB1	(84)
*In vitro*	SKOV3 cells (Taxol-resistant)	Bicalutamide	↑Cytotoxicity ↓ABCB1	(84)
*In vitro*	HeyA8, OVCAR-3, SKOV3ip1 cells	AR cDNA	↑ABCG2, ABCG2 efflux ability, Cell growth, Survival	(85)
*In vitro*	HeyA8, OVCAR-3, SKOV3ip1 cells	shAR	↓ABCG2, Cell growth, Survival	(85)
*In vitro*	HeyA8, OVCAR-3, SKOV3ip1 cells	Paclitaxel	↑AR, ABCG2	(85)
*In vitro*	OVCAR-3 cells (Paclitaxel-resistant)	Paclitaxel+ ASC-J9	↓Colony number, Survival, AR	(85)
*In vivo*	Athymic nude mice (OVCAR-3 paclitaxel-resistant cell) xenografts	Paclitaxel+ ASC-J9	↓Tumor volume	(85)
*In vitro*	SKOV3, OV90, OVCAR-3, COV362.4, OVCAR-8, A2780 cells	DHT	↑AR activity	(86)
*In vitro*	SKOV3, OV90, OVCAR-3, COV362.4, OVCAR-8, A2780 cells	Metformin	↑AR activity ↓PI3K	(86)
*In vitro*	SKOV3 cells (TLR4HA OE)	Taxol	↑TLR4, AR, IL-6	(87)
*In vitro*	SKOV3 cells (Taxol resistant)	Taxol	↑TLR4, AR, IL-6	(87)
*In vitro*	SKOV3 (Taxol-resistant)	Paclitaxel	↑AR, H1.0	(88)
*In vitro*	SKOV3 (Taxol-resistant cells with AR knockdown)	Paclitaxel	↓AR, H1.0, ABCB1, ABCG2	(88)
Estrogen Receptor				
*In vitro*	PE04 cells	E2	↑Cell growth	(89)
*In vitro*	PE04 cells	4-hydroxitamoxifen	↓Cell growth	(89)
*In vitro*	PEO4 cells	E2	↑Cell growth	(90)
*In vitro*	PEO4 cells	Tamoxifen	↓Cell growth, Colony formation	(90)
*In vivo*	BALB/c nude mice (OVA-5) xenograft	17-β-estradiol	↑Tumor growth	(76)
*In vivo*	BALB/c nude mice (OVA-5) xenograft	5-α-testosterone	↑Tumor growth	(76)
*In vivo*	Ovariectomized nude mice (JOHYL-1 cells) xenograft	Estrogen	↑ER, Tumor volume ↓Volume doubling time	(91)
*In vitro*	BG-1 cells	Estradiol	↑Procathepsin D, Cell growth	(92)
*In vitro*	PEO4 cells	17-β-estradiol	↑Cell growth, Cell number	(93)
*In vivo*	Nude mice (PE04 cells) xenograft	17-β-estradiol	↓Tumor growth	(93)
*In vivo*	Nude mice (PEO4 cells) xenograft	Tamoxifen	↓Tumor growth, ER	(93)
*In vitro*	A2780 cells	ICI 182, 780	↑Apoptosis, G1 arrest, DNA fragmentation, Hyper-aggregation of chromatin ↓Cell growth	(94)
*In vitro*	A2780 cells	Tamoxifen	↑Apoptosis, G1 arrest, DNA fragmentation, Chromatin clumping ↓Cell growth	(94)
*In vitro*	BG-1 cells	Estradiol	↑Cell proliferation ↓Fibronectin-induced migration	(95)
*In vitro*	BG-1, SKOV3 cells	Estradiol+ Fibronectin+ Fibulin-1	↓Cell motility	(95)
*In vitro*	NIH: OVCAR-3, CAOV3 cells	Genistein or daidzein	↑TGF-β1 production ↓DNA synthesis, Cell viability, IL-6 synthesis	(96)
*In vitro*	BG-1 cells	E2	↑SDF-1α, SDF-1β, Cell proliferation	(97)
*In vitro*	BG-1 cells	ICI 182, 780	↓SDF-1, Cell proliferation	(97)
*In vitro*	BG-1 cells	Estradiol+ ER (OE)	↑Cell proliferation, Micronucleus formation	(98)
*In vitro*	BG-1 cells	Hydrotamoxifen+ ER (OE)	↓Cell proliferation, Micronucleus formation	(98)
*In vitro*	BG-1 cells	ICI 182, 720	↑E-cadherin ↓Cell proliferation	(99)
*In vitro*	BG-1 cells	E2	↑Cell migration, EMT, Snail, Slug ↓E-cadherin	(99)
*In vitro*	Br-1 cells	E2	↓C3, CLU, COL6A1, DLC1, NME1, NRIP1, PTEN, RAC2, S100A2, ERBB2, ID2	(100)
*In vitro*	Br-1 cells	Genistein	↓C3, CLU, COL6A1, DLC1, NME1, NRIP1, PTEN, RAC2, S100A2, ERBB2, ID2, LCN2, PHB2, HMGB1	(100)
*In vitro*	Oy-1 cells	E2 and/or Genistein	↑MK167, SERPINB5, SLC7A5, CDK1NA, LCN2, PLAU, PHB2, CTSB, EGLN2, ERBB2, HMGB1, ID2, ITGB4, TOP2A	(100)
*In vitro*	OAW-42 cells	Tamoxifen	↓Cell proliferation	(101)
*In vivo*	Athymic nude mice (PE04 cells) xenograft	E2	↑Tumor growth and size, Lymph node metastasis, PR	(102)
*In vitro*	BG-1 cells	Di-n-butyl phthalate, E2, hexabromocyclodecane	↑Cell proliferation, Cyclin D, Cdk-4	(103)
*In vitro*	IOSE-385, OVCAR-3 cells	17-β-estradiol, 2-hydroxy estradiol, 4-hydroxy estradiol, 2-methoxy estradiol, 4-methoxy estradiol	↑Cell proliferation	(104)
*In vitro*	OVCAR-3, SKOV3, OVCA-432, IOSE-385 cells	Ascorbic acid	↓Cell number	(104)
*In vitro*	IOSE-385, OVCAR-3, OVCA-432 cells	Ascorbic acid+ Estradiol 17β/2-hydroxy estradiol/4- hydroxy estradiol/2-methoxy estradio/4-methoxy estradiol	↑Cell proliferation	(104)
*In vitro*	BG-1 cells	E2, Methoxychlor, Trichlosan	↑Cell growth, Cyclin D1 ↓p21, Bax	(105)
*In vitro*	SKOV3, OV2008 cells	E2	↑Cell viability	(105)
*In vitro*	SKOV3 cells	MPP+ DPN	↑Cell viability ↓p-Akt	(106)
*In vitro*	SKOV3 cells	E2+ BTB	↓ER transactivation, Cell growth	(107)
*In vitro*	OVCAR-3 cells	E2	↑Cell growth, ROS, NO, Cell viability	(108)
*In vitro*	OVCAR-3 cells	E2+ICI 182, 780/N-acetyl-L-cysteine	↓ E2-induced ROS production	(108)
*In vitro*	BG-1 cells	Fenhexamid, E2	↑Cell proliferation, Cyclin D1 and E, Cathepsin D	(109)
*In vivo*	BALB/c nude female mice (BG-1) xenograft	E2, Cyprodinil	↑Tumor volume, PCNA, Cathepsin D	(109)
*In vitro*	BG-1 cells	Bisphenol A/E2/Nonylphenol	↑Snail, Slug, Vimentin, MMP-9, Cell migration ↓E-cadherin	(110)
*In vitro*	BG-1 cells	Bisphenol A/E2/Nonylphenol+ ICI 182, 780	↑E-cadherin, pSmad3 ↓Snail, Slug, Vimentin, MMP-9, Cell migration	(110)
*In vitro*	OVCAR-3, SKOV3 cells	Bisphenol A	↑VEGF-R2, VEGF-A	(111)
*In vitro*	BG-1 cells	E2, Benzophenone-1, 4-tert-octylphenol	↑Cell migration, Snail, Slug, N-cadherin ↓E-cadherin	(112)
*In vitro*	BG-1 cells	Benzophenone-1 or 4-tert-octylphenol+ ICI 182, 780	↓Cell migration, Snail, Slug, N-cadherin ↑E-cadherin	(112)
*In vitro*	OVCAR-3 cells	E2	↑CXCR7, Snail, Slug, N-cadherin ↓E-cadherin	(7)
*In vitro*	OVCAR-3 cells	ICI 182,780	↓CXCR7, ER degradation	(7)
*In vitro*	SKOV3, A2780CP cells	Genistein, Daidzein	↓Cell proliferation, Migration, Invasion	(113)
*In vitro*	SKOV3, A2780CP, OVCAR-3 cells	ERB-041	↓Migration, Invasion, Proliferation	(113)
*In vitro*	SKOV3 cells	siERβ	↑Migration, Invasion	(113)
*In vitro*	SKOV3 cells	Genistein	↑Apoptosis, S and G2/M cell cycle arrest	(113)
*In vitro*	SKOV3 cells	Daidzein, ERB-041	↑G1 cell cycle arrest	(113)
*In vitro*	SKOV3 cells	Genistein, daidzein, ERB-041	↑Apoptosis ↓FAK, PI3K/Akt/GSK signaling	(113)
*In vitro*	OVCAR-3 cells	Genistein, daidzein, ERB-041	↓Sphere formation	(113)
*In vitro*	CAOV3 cells	17β-E2+ siRNA-LINC00511	↑Apoptosis ↓Migration, Invasion, Cell viability	(114)
*In vivo*	Nude mice (CAOV3 cells) xenograft	siRNA-LINC00511	↓Tumor volume, Ki67	(114)
*In vitro*	OVCAR-3, SKOV3 cells	Cadmium chloride+ ICI182,780	↓Cell growth, ERα, ERβ, p-ERK1/2, c-fos, c-jun, FOXO3a	(115)
*In vitro*	OVCAR-3, SKOV3 cells	Trametinib+ tamoxifen+ estrogen	↓p-ERK	(116)
*In vitro*	OVCAR-3, SKOV3 cells	Trametinib, erlotinib	↓Cell growth, p-AKT, p-ERK, p-ER	(116)
*In vitro*	OVCAR-3 cells	Bisphenol A	↑Cell proliferation, Migration, Invasion, Adhesion, MMP-2&-9, ICAM-1	(117)
*In vitro*	SKOV3 cells	miR-26b OE	↑E-cadherin, G0/G1 cell cycle arrest ↓Cell proliferation, Migration, Invasion, Vimentin, Snail, Slug, MMP-2&-9	(118)
*In vitro*	CAOV3 cells	miR-26b inhibitor	↑Migration, Invasion, Vimentin, Snail, Slug, MMP-2&-9 ↓E-cadherin	(118)
*In vitro*	SKOV3 cells	siERα	↓Cell proliferation, Migration, Invasion, Vimentin, Snail, Slug, MMP-2&-9, S-phase cells ↑E-cadherin, G0/G1 arrest	(118)
*In vivo*	Nude mice (SKOV3 cells) xenograft	OE miR-26b	↓Tumor volume, Tumor weight, ERα, Vimentin, Snail, Slug, MMP-2, MMP-9 ↑E-cadherin	(118)
*In vitro*	OV2008, PEO1, OVCAR-4, OVCAR-3, Kuramochi OV39 organoid	OSU-ERb-12	↓Relative cell viability	(119)
*In vitro*	PEO1, OVCAR-3, OVCAR-4 cells	OSU-ERb-12	↑E-cadherin ↓EMT, Snail	(119)
*In vitro*	OVCAR-3, OVCAR-4 cells	OSU-ERb-12	↓ALDH+ cells, Sphere formation	(119)
*In vitro*	OVCAR-3, PEO1 cells	OSU-ERb-12	↓CSC population, CCND1, NRIP1	(119)
*In vivo*	NSG mice (human ovarian papillary serous adenocarcinoma tissues) xenografts	OSU-ERb-12	↓Tumor volume	(119)
ERα				
*In vitro*	OV266 cells	Suramin	↑Cell growth	(120)
*In vitro*	BG-1 cells	PPT	↓E-cadherin	(99)
*In vitro*	BG-1 cells	siERα	↑E-cadherin	(99)
*In vitro*	SKOV3 cells	ERα (OE)	↓E-cadherin	(99)
*In vitro*	BG-1 cells	Leptin	↑Cell growth, ERα nuclear expression, p-STAT3, p-Akt	(121)
*In vitro*	OVCAR-3, A2780 cells	Leptin+ pCMV5- ERα	↑Cell growth	(121)
*In vitro*	OVCAR-3, A2780 cells	Leptin+ pCMV5- ERα+ ICI 182,780	↓Cell growth	(121)
*In vitro*	BG-1 cells	Leptin+ siERα	↓Cell growth	(121)
*In vitro*	BG-1 cells	Leptin+ siERα+ E2	↓Cell growth	(121)
*In vitro*	BG-1 cells	Leptin+ MPP	↓Cell growth	(121)
*In vitro*	BG-1 cells	E2	↑Cell proliferation ↓ERα, FOXA1, GATA3, MEF2C	(122)
*In vitro*	BG-1 cells	4-hydroxytamoxifen	↑ERα, pS2, FOXA1 ↓CCNA, CCNB1, MEF2C	(122)
*In vitro*	BG-1 cells	Fulvestrant	↑CCNB2 ↓Cell growth, ERα expression, CCND, pS2, Fibulin-1, RIP140 1	(122)
*In vitro*	BG-1, PE01R cells	E2	↑Nuclear ERα, Src, ER/Src binding, Cell proliferation	(123)
*In vitro*	PE01R cells	PPT or THC	↑Cell proliferation	(123)
*In vitro*	BG-1, PE01R cells	Saracatinib+ fulvestrant	↑p27, LC3-II, cleaved PARP, Apoptosis ↓Cyclin E-cdk2, Cell cycle progression, c-Myc, p-Src, FOSL1	(123)
*In vivo*	Female BALB/c nude mice (PE01R cells) xenograft	E2	↑Tumor growth	(123)
*In vivo*	Female BALB/c nude mice (PE01R cells) xenograft	Saracatinib+ fulvestrant	↓Tumor growth	(123)
*In vitro*	BG-1 cells	E2	↑Cell growth, Cyclin D1 ↓p21	(124)
*In vitro*	BG-1 cells	Benzophenone-1	↑Cell growth, Cyclin D1	(124)
*In vitro*	BG-1 cells	ICI 182, 780+ E2/Benzophenone-1	↓Cell growth	(124)
*In vivo*	Female BALB/c nude mice (BG-1 cells) xenograft	Benzophenone-1, E2	↑Tumor mass formation, BrdUrd positive nuclei, Cyclin D1 ↓p21	(124)
*In vitro*	SKOV3, OV2008 cells (Supplemented with charcoal dextran treated FBS)	MPP	↓Cell growth	(106)
*In vitro*	SKOV3, OV2008 cells (Supplemented with charcoal dextran treated FBS)	PPT	↑Cell growth	(106)
*In vitro*	SKOV3 cells	MPP	↓p-Akt	(106)
*In vivo*	BALB/c nude mice (SKOV3) xenograft	MPP	↓Tumor size and volume	(106)
*In vitro*	SKOV3 cells	BTB	↓c-Myc, Cyclin D1, E2F1, and TERT	(107)
*In vitro*	PA-1 cells	E2	↑Cell migration, Invasion, p-ERK, CD133^+^ cells, N-cadherin, Slug, miR-21, miR-99a ↓E-cadherin	(125)
*In vitro*	SKOV3 cells	ERα (OE)+ E2	↑Cell proliferation, Semaphorin D	(126)
*In vitro*	OVCAR-3 cells	Bisphenol A	↑Cell viability, proliferation, glycolysis, intracellular ATP, Pyruvic acid, Lactate production	(127)
*In vitro*	OVCAR-3 cells	siERα	↓Cell proliferation	(127)
*In vitro*	OVCAR-3 cells	PPT	↑CXCR7	(7)
*In vitro*	TOV21G cells	E2	↑CXCR7, PR	(7)
*In vitro*	SKOV3 cells	Leptin	↑ERα, Cell growth, Invasion, MMP-7&-9, upA	(128)
*In vitro*	SKOV3 cells	Leptin+ siOB-Rb	↓ERα	(128)
*In vitro*	SKOV3 cells	Leptin+ ICI 182,780	↓Cell growth, Invasion	(128)
*In vitro*	SKOV3 cells	Leptin+ siERα	↓MMP-9	(128)
*In vitro*	SKOV3 cells	Leptin+ PPT	↑Cell growth	(128)
*In vitro*	OVCAR-3, SKOV3, OV-90, COV318 cells	4-Hydroxy-Tamoxifen	↓Cell viability	(129)
*In vitro*	OVCAR-3, SKOV3, OV-90, COV318 cells	Gatipotuzumab+ 4-OHT	↓Cell viability	(129)
*In vitro*	OVCAR-3, SKOV3, OV-90, COV318 cells	Gatipotuzumab	↓Cell viability	(129)
*In vitro*	SKOV3 cells	Benzophenone-1	↑Nuclear β-catenin, Proliferation, Invasion, Migration, MMP-9 ↓ZO-1	(130)
*In vitro*	OVCAR-3 cells	Bisphenol A+ MPP	↓Cell proliferation, MMP-2&-9, ICAM-1	(117)
ERβ				
*In vitro*	SKOV3 cells (Transfected with ERβ1 isoforms)	E2+ ERβ1 cDNA	↑p21, Fibulin-1c, Caspase-3&-7 ↓Cell proliferation	(131)
*In vitro*	SKOV3 cells	siERβ1	↑Cell proliferation	(131)
*In vitro*	SKOV3 cells	E2+ siERβ1	↑Cell proliferation ↓Caspase-3&-7	(131)
*In vitro*	BG-1 cells	DPN	↑E-cadherin	(99)
*In vitro*	SKOV3 cells	siERβ+ E2	↓E-cadherin	(99)
*In vitro*	ES-2 cells	ERβ cDNA+ E2	↓Cell proliferation, Migration, cyclin D1	(132)
*In vitro*	PE01R cells	DPN	↓Cell proliferation	(123)
*In vitro*	PE01R cells	PHTPP+ E2	↑Cell proliferation	(123)
*In vitro*	SKOV3, OV2008 cells (Charcoal dextran treated)	DPN	↓Cell growth	(106)
*In vitro*	SKOV3, OV2008 cells (Charcoal dextran treated)	PHTPP	↑Cell growth	(106)
*In vitro*	SKOV3 cells	DPN	↓p-Akt	(106)
*In vivo*	BALB/c nude mice (SKOV3) xenograft	DPN	↓Tumor size and volume	(106)
*In vitro*	SKOV3, BG-1, SKOV3 (Taxol resistant), ES2 (Cisplatin resistant) cells	Liquiritigenin	↓Cell viability, Migration and invasion, NF-κB	(133)
*In vitro*	ES2, SKOV3, SKOV3 (Taxol resistant) cells	Liquiritigenin, S-equol	↑Apoptosis, Caspase-3&-7	(133)
*In vitro*	ES2, SKOV3 cells	Liquiritigenin+ paclitaxel	↑Sensitivity to paclitaxel ↓Cell viability	(133)
*In vitro*	ES2, SKOV3 cells	Liquiritigenin+ cisplatin	↑Sensitivity to cisplatin ↓Cell viability	(133)
*In vitro*	SKOV3 cells (ERβ positive)	ERβ OE+ E2	↓Cell proliferation, Semaphorin D	(126)
*In vitro*	TOV-21G cells	ERβ2 cDNA	↑Cell migration and invasion	(134)
*In vitro*	ES-2, OVCA420 cells	ERβ5 cDNA	↑Cell proliferation, Migration and invasion, FAK	(134)
*In vitro*	TOV21G cells	ERβ OE+ DPN	↑CXCR7	(7)
*In vitro*	A2780 cells	Aconitine	↑Mitochondrial apoptosis, DNA damage, ERβ, prolyl hydroxylase 2, p53, Bax, apoptotic peptidase activating factor 1, cytochrome C, cleaved caspase−3&-9, cleaved PARP ↓Cell viability, colony formation and motility, VEGF, HIF-1α, Bcl-2, Bcl-xl, ATM serine threonine kinase, MMP-2&-9, Cell migration	(135)
*In vitro*	IGROV1 cells	3-([2-chloro-1-(4-chlorobenzyl)-5-methoxy-6-methyl-1H-indol-3-yl] methylene)-5-hydroxy-6-methyl-1,3-dihydro-2H-indol-2-one	↑ESR2, G_0_/G_1_ phase arrest, P16, ERβ1 ↓Cell proliferation, CCND1, MYC, ERβ2	(136)
*In vitro*	SKOV3 (ALDH+), A2780 (ALDH+) cells	LY500307	↑Apoptosis, FDXR, p21/CDKN1A, Cleaved PARP, Caspase 3, G2/M cell cycle arrest ↓Cell viability, Sphere formation, Self-renewal, Invasion, SOX2, Oct4, DOK3, PDK1, ANKRD36, AGER, SLC26A10, CFH, DNHD1, MYBL1, PRRT2, HK2, STC2, CCDC18	(137)
*In vivo*	OCSCs orthotopic mice (SKOV3 (ALDH^+^) cells) xenograft	LY500307	↓Tumor initiating capacity of OCSCs	(137)
Estrogen-related Receptor				
*In vitro*	OVCAR-3 cells	ERRα cDNA	↑EMT, N-cadherin, Snail ↓E-cadherin	(138)
*In vitro*	SKOV3 cells	siERRα	↓EMT, N-cadherin, Snail, Nanog, Bmi-1, Oct-4, Number of spheres ↑E-cadherin, miR-141, miR-200a, miR-200b, miR-200c	(138)
*In vivo*	NOD/SCID mice (siERRα-SKOV3 cells) xenograft	–	↓Ascites volume, Tumor nodule size, Tumor nodule number	(138)
*In vitro*	ES-2, SKOV3 cells	Cordycepin	↓Cell viability, Mitochondrial activity	(139)
*In vitro*	OVCAR-3 cells	Cordycepin	↑E-cadherin, Fis-1 ↓ERRα, Cell viability, Vimentin, EMT, Mitochondrial activity, Mfn-1, Mfn-2, Migration	(139)
*In vitro*	OVCAR-3 cells	Cordycepin+ siERRα	↑Mitochondrial fission ↓EMT, Migration, Mitochondrial membrane potential, Mitochondrial activity	(139)
Glucocorticoid Receptor				
*In vitro*	3AO cells	Dexamethasone	↑Alkaline phosphatase ↓Cell proliferation, CA125, GR	(140)
*In vitro*	3AO cells	Dexamethasone	↑Alkaline phosphatase ↓GR binding activity, GR	(141)
*In vitro*	SKOV3, HeyA8 cells	Dexamethasone+ Paclitaxel	↑SGK1, MKP1/DUSP1	(142)
*In vitro*	SKOV3, HeyA8 cells	Dexamethasone+ Carboplatin/Gemcitabine	↑GR, Survival, SGK1, MKP1/DUSP1 ↓Cell death	(143)
*In vitro*	SKOV3, HeyA8, Monty-1 cells	Dexamethasone+ Mifepristone/CORT125134	↓SGK1, MKP1	(143)
*In vivo*	HGS-OvCa tumor xenograft mouse model	Carboplatin/Gemcitabine/Mifepristone or Gemcitabine/Carboplatin	↓Tumor weight	(143)
*In vitro*	SKOV3, HO-8910 cells	Dexamethasone	↑Fibronectin, Cell adhesion, Survival, PI3K/Akt pathway, MUC1	(144)
*In vivo*	OVCAR-5 xenograft mice model	ORIC-101 + Gemcitabine + Carboplatin + Cortisol	↑Response to chemotherapy ↓Tumor volume	(145)
*In vitro*	OVCAR-5 cells	Dexamethasone+ Paclitaxel	↑Cell viability ↓Cytotoxicity of paclitaxel	(146)
*In vivo*	MIA PaCa-2 xenograft	Relacorilant+Paclitaxel	↓Tumor volume	(146)
NR1D1				
*In vitro*	OVCAR-3 cells	NR1D1 OE	↑G1 phase arrest, Apoptosis, Caspase-3&-9, SOCS3 ↓Cell proliferation, PCNA, Cyclin D, Cyclin E, JAK/STAT3 pathway	(147)
*In vivo*	OVCAR-3 xenograft mice model	NR1D1 OE	↑SOCS3 ↓Tumor volume, p-JAK1, p-JAK2, p-STAT3	(147)
Nurr77/NR4A1/TR3				
*In vitro*	PA-1 cells	Vitamin K2	↑TR3, Apoptosis, Cytochrome C ↓ Cell growth	(148)
*In vitro*	OVCAR-3, OVCAR-8 cells	Cisplatin	↑TR3, Cleaved PARP, Apoptosis, JNK ↓Akt pathway, Cell growth	(149)
*In vitro*	OVCAR-8 cells	Cisplatin+ shTR3	↑p21 ↓Cell growth	(149)
*In vivo*	Athymic Nude-Foxn1^nu^ mice (OVCAR-8) xenograft	Cisplatin+ shTR3	↓Tumor growth, cleaved Caspase-3	(149)
Peroxisome Proliferator-Activated Receptor				
*In vitro*	A2780, OVCAR3, OVCAR-5, OVCAR-8, SKOV3, IGROV1 cells	Ciglitazone	↓Cell growth	(67)
*In vitro*	A2780 cells	Ciglitazone	↑Apoptosis, p53, p21, Bax, Caspase-3, PTEN ↓Cell growth, Survival, G1 phase arrest, Survivin, Cyclin D1, c-Myc	(67)
*In vitro*	A2780, OVCAR-5, OVCAR-8 cells	Ciglitazone	↓Colony formation	(67)
*In vitro*	SKOV3 cells	1,1-Bis(3’-indolyl)-1-(p-t-butylphenyl) methane	↑p21, Apoptosis, Glucose-related protein 78, G0-G1 phase arrest ↓Cell proliferation, Cell survival, S phase, p-Rb, Cyclin D1	(150)
*In vitro*	OVCAR-3 cells	Clofibric acid	↑ Carbonyl reductase, PPARα, BE, PGE_2_ ↓ Cell growth	(151)
*In vitro*	DISS cells	Clofibric acid	↓ Cell growth	(151)
*In vivo*	Cancer-bearing mouse model (OVCAR-3) and a cancerous peritonitis mouse model (DISS)	Clofibric acid	↑ Survival, Apoptosis, Carbonyl reductase ↓Tumor volume, Tumor weight, Microvessel density, Microsomal PGE Synthase, PGE_2_, VEGF	(151)
*In vivo*	Cancer-bearing mouse model (OVCAR-3) and peritoneal carcinomatosa mouse model (DISS)	Meloxicam	↑Apoptosis ↓Tumor growth, PGE_2_, Microvessel density, COX-2, VEGF	(152)
*In vivo*	Cancer-bearing mouse model (OVCAR-3) and peritoneal carcinomatosa mouse model (DISS)	Ciglitazone	↑Apoptosis, PPARγ ↓Tumor growth, PGE_2_, Microsomal prostaglandin (PG) E synthase, Microvessel density, VEGF	(152)
*In vitro*	HO-8910 cells	PGC-1α cDNA	↑Apoptosis, Cell shrinkage, Condensed chromatin, Bax, Bak1, Hrk, Bcl-2l11, Bcl-2l1, Bcl-2l13, CIDE3, CIDEA, DFFA, Cytochrome c ↓Bcl-2, BIRC3, BIRC5, BIRC1	(153)
*In vivo*	Nude mouse (OVCAR-3 cells) xenograft	Clofibric acid or Pioglitazone	↑Apoptosis ↓Tumor volume, Tumor weight, VEGF, Microvessel density, AP-1, COX-2	(154)
*In vivo*	Nude mouse (OVCAR-3 cells) xenograft	Clofibric acid+ Pioglitazone	↑Apoptosis ↓Tumor volume, Tumor weight, VEGF, Microvessel density, AP-1, COX-2	(154)
*In vivo*	Peritoneal carcinomatosa mouse model (DISS cells)	Clofibric acid+ Pioglitazone	↑Survival	(154)
*In vitro*	A2780 cells	Troglitazone	↓Cell viability	(155)
*In vitro*	A2780 cells	Clofribic acid	↓Cell viability	(155)
*In vitro*	A2780 cells	Clofibric acid+ Clioquinol	↑Apoptosis, Necrosis ↓Cell viability	(155)
*In vitro*	A2780 cells	Clioquinol+ DHA+ GW9662 (10μM)	↑Cell viability ↓Cytotoxicity	(155)
*In vitro*	A2780 cells	Clioquinol+ DHA+ GW6471	↑Cell viability	(155)
*In vitro*	OVCAR-3 cells	Ciglitazone	↑Apoptosis ↓Glucose uptake	(156)
*In vitro*	A2780 cells	Ciglitazone	↑Apoptosis, p-AMPK ↓Glucose uptake, GLUT-1, Sp-1, β-catenin	(156)
*In vitro*	A2780, OVCAR-3 cells	Ciglitazone + siGLUT-1	↑Apoptosis	(156)
*In vivo*	NOD-SCID IL2Rgamma^null^ mouse (A2780 cells) xenograft	Ciglitazone	↓Tumor volume	(156)
*In vitro*	HEY cells	Telmisartan	↑Apoptosis, Caspase-3, PPARγ ↓Cell growth, MMP-9	(157)
*In vitro*	OVCAR-3 cells	BPA/TBBPA/TCBPA	↑Apelin expression	(158)
*In vitro*	COV434 cells	Rosiglitazone	↓Chemerin expression, RARRESR2	(51)
*In vitro*	COV434 cells	BPA/TBBPA/TCBPA	↓Chemerin	(51)
*In vitro*	SKOV3 cells	Oroxylin-A	↑Apoptosis, PPARγ, PGRMC2 ↓Cell proliferation, Migration, PGRMC1	(159)
*In vitro*	SKOV3, A2780 cells	MEHP	↑Migration, Invasion, Vimentin, N-cadherin, Slug, PIK3CA ↓ZO-1, E-cadherin	(160)
*In vitro*	SKOV3, A2780 cells	MEHP+ siPPARα	↓PIK3CA	(160)
*In vivo*	Nude mice (SKOV3) xenograft	MEHP	↑Metastatic tumor nodules, Metastatic tumor weight	(160)
Progesterone Receptor				
*In vivo*	Ovariectomized nude mice (JOHYL-1 cells) xenograft	Estrogen+ Progesterone	↑PR, Volume doubling time ↓Tumor growth	(91)
*In vivo*	Nude mice (PEO4 cells) xenograft	Tamoxifen	↓Tumor growth, PR	(93)
*In vivo*	Nude mice (PE04 cells) xenograft	Megestrol acetate	↓Tumor volume	(161)
*In vitro*	Ovarian tumor cells	β-estradiol or Testosterone	↑PR ↓Cell survival	(162)
*In vitro*	OVCA 432, OVCA 429 cells	Estrone or 17-β-estradiol	↓PR	(68)
*In vitro*	OVCA 432, OVCA 429 cells	P4	↑Caspase-3 ↓Cell growth	(68)
*In vitro*	OVCA 432, OVCA 429 cells	P4+ ICI	↑Caspase-3 ↓Cell growth	(68)
*In vitro*	SKOV cells	cAMP	↑PR-B, p21, p27, Cell senescence, G0/G1 fraction ↓p-Rb, Cell growth, Colony formation, S-phase fraction	(163)
*In vitro*	SKOV cells	PR-B OE	↓Cell growth	(163)
*In vitro*	SKOV cells	MPA	↑p27 ↓Cell growth	(163)
*In vitro*	SKOV cells	PR-B OE+ MPA	↑p21, p27 ↓Cell growth	(163)
*In vitro*	Cisplatin-treated HO-8910 cells	Progesterone	↑S-Phase cells, partially restored migration, Invasion, Akt signaling ↓Cisplatin-induced inhibition of proliferation, PGRMC1, PGR	(164)
*In vitro*	ES-2 cells (transfected with GFP-tagged PR-B)	R5020	↑PR transcriptional activity, overall size of nuclei, SAβGal-positive cells, G0/G1 cells, p21, FOXO1, PR ↓% of total colonies	(165)
*In vitro*	PEO4 cells	R5020	↑SAβGal-positive cells	(165)
*In vitro*	PR+ PEO4 cells	R5020	↑p21	(165)
*In vitro*	progestin-treated PR+ PEO4 cells	R5020	↑FOXO1	(165)
*In vitro*	NIH: OVCAR-3, ES-2 cells	P4	↑ADAMTS 1 and 4	(166)
*In vitro*	OVCAR-3 cells	P4+ CAL	↑CYP24A1 ↓Cell viability	(167)
*In vitro*	OVCAR-3 (PR-transfected)	P4+ CAL	↓Cell viability, CYP24A1	(167)
*In vitro*	OVCAR-5 cells	P4+ CAL	↓Cell viability	(167)
*Ex vivo*	Endometrioid ovarian carcinoma tissues	P4	↓Cell number	(168)
*In vitro*	OVCAR-3, OC-3-VGH cells	PR-B cDNA+ Cisplatin	↓Cell viability ↑Apoptosis, Sensitivity to cisplatin	(50)
*In vitro*	OVCAR-3-PR-B, OC-3-VGH-PRB cells	P4+ Cisplatin	↑Sensitivity to cisplatin ↓Cell viability	(50)
Retinoic Acid Receptor				
*In vitro*	HOC-7, HEY, H134, TR 170, SKOV3, Ca-OV3, NIH: OVCAR-3, PA-1 cells	All trans-RA, cis-RA, TTNBP, TTNBP ethylester, TTNN	↓Cell growth	(169)
*In vitro*	OVCAR-3 cells	All trans-RA	↓Cell growth	(170)
*In vitro*	OVCAR-8 cells	8-Cl-cAMP+9-cis, 13-cis or all trans-RA	↓Cell growth	(170)
*In vitro*	OVCAR-3 cells	8-Cl-cAMP+9-cis, 13-cis or all trans-RA	↑Caspase 3, Apoptosis, DNA fragmentation, RARβ, PARP cleavage ↓Colony formation	(170)
*In vitro*	OVCAR-8 cells	8-Cl-cAMP+9-cis, 13-cis or all trans-RA	↑Caspase 3, Apoptosis, DNA fragmentation, PARP cleavage, Free nucleosomes, RARβ ↓Colony formation	(170)
*In vitro*	CAOV3, SKOV3, SK-γx, CA-R269Q cells	AHPN/CD437	↑Apoptosis ↓Cell number	(171)
RARα				
*In vitro*	HOC-7, HEY, H134, TR 170, SKOV3, CAOV3, NIH: OVCAR-3, PA-1 cells	All trans-RA	↑RARα	(169)
*In vitro*	CAOV3 cells	RA	↑RARα ↓Cell growth, CAT activity	(56)
*In vitro*	CAOV3, SKOV3 cells	RA	↑DR-5 binding activity	(56)
*In vitro*	SKOV3 cells (RARα OE)	RA	↑Growth inhibition	(56)
*In vitro*	A2780, IGROV-1 cells	4HPR	↓Sensitivity	(172)
*In vivo*	A2780 RARα-transfected clones injected mice	4HPR	↑RARα ↓Takes of clones Tumor latency delayed	(172)
*In vitro*	A2780 cells	4MPR	No cell growth inhibition	(172)
RARβ				
*In vitro*	HOC-7, HEY, H134, TR 170, SKOV3, CAOV3, NIH: OVCAR-3, PA-1 cells	All trans-RA, cis-RA, TTNBP, TTNBP ethylester, TTNN	↑RARβ	(169)
*In vitro*	AD10 cells	4HPR	↑RARβ ↓Anchorage-dependent cell growth	(173)
*In vitro*	UCI101, 222, CP70 cells	4HPR	↑RARβ ↓Cell growth	(173)
*In vitro*	SKOV3 cells (RARβ OE)	RA	↓Cell growth	(56)
*In vitro*	A2780, IGROV-1 cells	4HPR	↑Sensitivity	(172)
*In vitro*	A2780 cells (RARβ-transfected clones)	4HPR	↑RARβ, Sensitivity to antiproliferative effect of 4HPR ↓Cell number	(172)
*In vivo*	A2780 RARβ-transfected clones injected mice	4HPR	↑RARβ ↓Tumor growth	(172)
*In vitro*	OVCAR-3, OVCAR-8	8-Cl-cAMP+All trans-RA	↑Apoptosis, Caspase-3, cleaved PARP, RARβ	(170)
*In vitro*	CAOV3, OVCAR-3, HOC-7 cells	PD153035	↑RARβ	(174)
*In vitro*	OVCAR-3 cells	PD153035	↑RARβ, Unmethylated promoter sequence (RARβ-M) ↓Methylated promoter sequence (RARβ-U)	(174)
RARγ				
*In vitro*	HOC-7, HEY, H134, TR 170, SKOV3, CAOV3, NIH: OVCAR-3, PA-1 cells	All trans-RA	↑RARγ	(169)
*In vitro*	SKOV3 cells	RA	↓Cell growth	(56)
*In vivo*	A2780 RARα-transfected clones injected mice	4HPR	↑RARγ	(172)
*In vitro*	SKOV3 cells (RARγ OE)	AHPN/CD437	↑Apoptotis	(171)
*In vitro*	A2780, SKOV3 cells	si-RARγ	↓Cell proliferation, Colony number	(49)
*In vitro*	A2780, SKOV3 cells	sh-RARγ	↓Cell proliferation, Colony number	(49)
*In vivo*	BALB/c-NU mice (A2780 cells) xenograft	sh-RARγ	↓Tumor weight, Tumor volume, RARγ, Ki-67, PCNA	(49)
Retinoid X Receptor				
*In vitro*	SK-γx cells (RXRα and RXRγ OE)	AHPN/CD437	↑Apoptosis ↓Cell number	(171)
*In vitro*	A2780 cells	Resveratrol	↑Apoptosis ↓Cell viability, Sirt1	(175)
*In vitro*	A2780 cells (Carboplatin-resistant)	Resveratrol	↑Apoptosis, RXRα	(175)
*In vitro*	UWB1.289 cells	Resveratrol	↓Cell viability	(175)
RXRα				
*In vitro*	CAOV3, SKOV3 cells	RA	↓RAR/RXR activity	(56)
*In vitro*	CAOV3 cells	RA	↑CAT activity, RXRα	(56)
Vitamin D Receptor				
*In vitro*	OVCAR-3 cells	1α,25−dihydroxy vitamin D3	↑GADD45 ↓Cell growth, Cell cycle arrest at G1/S and G2/M, Cdc2 kinase, Cyclin B1	(176)
*In vitro*	SKOV3 cells	MT19c	↑MAPK, Apoptosis, cleaved Caspase 3, 9, cleaved PARP1, p-p38, P-SAPK/JNK, p16, p21, p27 ↓Cell proliferation, IRS-1/2 signaling, pERK1/2, Cyclin D1, D3, D4	(177)
*In vitro*	OVCA 420, OVCA 429 cells	Calcitriol	↓CXCL1, CXCL2, NF-κB, p-IκBα	(178)
*In vitro*	SKOV3 cells	MT19c	↓FASN, ACC, malonyl CoA carboxylase	(179)
*In vivo*	Nude mice (SKOV3 cells) xenograft	MT19c	↓Ascites volume, Animal weight, Tumor size, Tumor nodule formation, FASN, ACC, malonyl CoA carboxylase	(179)
*In vitro*	A2780 cells	1α,25−dihydroxy vitamin D3	↑Apoptosis ↓Migration, Adhesion to fibronectin	(180)
*In vivo*	Immunodeficient mouse (A2780 cells) xenograft	1α,25−dihydroxy vitamin D3	↓Metastasis, Seeding efficiency, Migration, Adhesion to fibronectin	(180)
*In vitro*	ES-2, TOV-21G cells	Calcitrol	↓Cell proliferation	(48)
*In vitro*	ES-2, TOV-21G cells	Calcitrol+ Progesterone	↑VDR ↓Cell proliferation, TGF-β1, TGF-βR1, TGF-βR2, SMAD2/3 and pSMAD2/3, CYP24A1	(48)
*In vitro*	Ovarian CSCs	1α,25−dihydroxy vitamin D3	↑VDR, β-catenin ↓Sphere forming rate, CD44, NANOG, OCT4, SOX2, Krüppel−like factor 4, Cyclin D1	(181)
*In vivo*	Nude mice xenograft (Ovarian CSCs)	1α,25−dihydroxy vitamin D3	↑β-catenin ↓Tumor volume, CD44, Stemness	(181)
*In vitro*	SKOV3 cells	1α,25−dihydroxy vitamin D3+IR radiation	↑Radiosensitivity, Apoptosis, VDR, NADPH oxidase ↓Sphere formation, Colony formation	(182)
*In vitro*	SKOV3 cells	shVDR	↑Colony formation ↓VDR	(182)
*In vivo*	Nude mice (OVCAR-8 cells) xenograft	1α,25−dihydroxy vitamin D3+IR radiation	↑Radiosensitivity, Survival ↓Tumor volume	(182)
*In vitro*	UT-OV-1(mucinous), UT-OV-3B (serous), UT-OV-4 (endometrioid) cells	Paclitaxel+ Carboplatin	↓Cell growth	(183)
*In vitro*	UT-OV-3B, UT-OV-4 cells	1,25-D3	↑VDR ↓Cell growth	(183)
*In vitro*	UT-OV-1, UT-OV-3B, UT-OV-4 cells	1,25-D3+ Paclitaxel+ Carboplatin	↑VDR ↓Cell growth	(183)
*In vitro*	SKOV3 cells	Calcitrol	↑VDR, Apoptosis ↓Cell survival and proliferation, VEGF	(184)
*In vitro*	SKOV3 cells	Calcitrol+ Cisplatin	↓Cell survival and proliferation, VEGF	(184)
*In vitro*	Patient-derived HGSOC cells (14433)	PRI-5201	↑VDR	(185)
*In vitro*	Patient-derived HGSOC cells (13781)	PRI-1907, PRI-5201, PRI-5202	↑VDR	(185)
*In vitro*	Patient-derived HGSOC cells (13781)	PRI-1906, PRI-1907, PRI-5201, PRI-5202, Calcitrol	↓Cell number, Cell viability	(185)
*In vitro*	Patient-derived HGSOC cells (14433)	PRI-1907, PRI-5201, PRI-5202	↓Cell number	(185)
*In vitro*	OvCa cells	Vitamin D+ TGFβ1	↑E-cadherin, ↓N-cadherin, MMP-2&-9, Slug, α-SMA	(186)

↓, Downregulation/inhibition; ↑, Upregulation/activation.

### 2.1 Androgen receptor

AR, also known as nuclear receptor subfamily 3, group C, gene 4 (NR3C4), located at Xq12, has been extensively studied as a TF that is dependent on ligand binding and is classified as a member of the NR superfamily (187–189). AR expression can be found in the regions of the body like the breast, cervix, endometrium, epididymis, fallopian tube, kidney, seminal vesicle, and testis (190).

Accumulating studies have revealed that AR expression has been upregulated, and androgen/AR signaling stimulated tumor growth and metastasis in OVC (86, 88, 191, 192). For instance, several studies in the past few decades have shown the effects of dihydrotestosterone (DHT), a synthetic androgen, in promoting AR translocation into the nucleus and increasing its expression in both OVC cells and *in vivo* models (76, 78–81). DHT acts as an agonist for AR, thereby inducing the proliferation and invasiveness of various OVC cells (78, 81). However, an intriguing study by Gogoi and colleagues showed that DHT and medroxyprogesterone acetate, an androgenic ligand, act by upregulating the inflammatory cytokines, IL-6 and IL-8 and downregulating the matrix metalloproteinases, MMP-2 and MMP-9 in OVC cells (79). Similarly, in another study, DHT increased cell proliferation in SKOV3 cells by elevating the IL-6 and IL-8 levels, which was reversed by treatment with an AR antagonist, flutamide (78). Further, the addition of R1881 (a synthetic anabolic androgenic steroid) caused cell growth and invasiveness by increasing the AR expression (82). It was observed that treatment with strong antagonists of AR, such as flutamide, OH-flutamide, anandron, and 3-β-hydroxysteroid dehydrogenase inhibitor, epostane reduced the survival and proliferation of OV166, OVC NOVA, and OV1225 cells adapted to DHT (75). Besides, OVCAR-3 and OSE2 cells, when treated with Casodex (bicalutamide), a potent anti-androgenic drug, inhibited cell proliferation and related genes by reducing AR level in the nucleus and resulted in cleavage of poly (ADP)-ribose polymerase (PARP) (80).

Taxol/paclitaxel, a mitotoxin, is well known for its growth-inhibitory properties of many cancers, including OVC. Yet, in patients, the effectiveness of this treatment is constrained by the acquisition of taxol resistance (txr). Kohli et al. reported that txr in OVC results from the upregulation of the linker histone H1.0 and the ABCB1 and ABCG2 drug transporters, followed by the recruitment of GCN5 and AR (88). Besides, Sun et al. demonstrated that FK506-binding protein 5 (FKBP5) was significantly upregulated in txr SKOV3 cells and knockdown of this protein resulted in increased sensitivity to taxol by downregulating several txr-related genes, including ABCB1, BMP5, FAT3, FGFR2, H1F0, SRCRB4D, STAG3, and TMPRSS15. Moreover, this study showed that FKBP5 forms a protein complex with AR which modulated the sensitivity of OVC cells towards taxol (82). Furthermore, the same group demonstrated that silencing of AR in txr OVC cells, resulted in increased apoptotic rate and decreased expression of AR mRNA and several multidrug resistance genes (83). In addition, they also found that suppression of ABCB1 gene helps in sensitizing txr OVC cells to paclitaxel or bicalutamide treatment (84). Another study demonstrated that AR expression and its nuclear translocation are influenced by the suppression of an AR molecular chaperone called small glutamine-rich tetratricopeptide repeat-containing protein alpha (SGTA) (81). Chung and colleagues demonstrated that overexpression of AR enhanced the cell growth, survival, and expression of the ABCG2 gene. This study also confirmed that the combinatorial treatment of paclitaxel with ASC-J9, an AR-degradation enhancer markedly reduced colony formation and survival of OVC cells (85).

Another study revealed the role of AR in promoting the growth of ovarian teratocarcinoma cells. AR overexpression improved the self-renewal capacity of cancer stem/progenitor cells (CSPCs). It was worth noting that the addition of DHT did not significantly affect cell growth or morphology, suggesting that these activities were carried out in a ligand-independent manner (57). Another study revealed the crosstalk between two NR signaling pathways where OVCAR-3 cells were treated with DHT and 1, 25-dihydroxy vitamin D3 [1,25(OH)2D3], which is an agonist of VDR. Notably, AR expression was enhanced by 1,25(OH)2D3 treatment but led to a reduction of growth in these cells (77). Another study demonstrated the treatment of txr OVC cells with androgen activated toll-like receptor 4 (TLR4) thereby and AR, thereby leading to AR-based txr. Hence, targeting TLR4 pathway can be used as an efficient strategy in overcoming txr in these cells (87). Collectively, the above studies illustrated that AR is upregulated in OVC, and therapeutic approaches targeting AR could be utilized for the prevention and management of this disease.

### 2.2 Estrogen receptor

ER, otherwise known as nuclear receptor subfamily 3 group A genes 1 (ERα) and 2 (ERβ) are positioned at 6q25.1 and 14q23.2, respectively (189, 193). ERα is predominantly present in reproductive tissues like the uterus and ovary and in the breast, kidney, bone, white adipose tissue, and liver. On the other hand, ERβ is expressed in various tissues such as the ovary, central nervous system, cardiovascular system, immune system, lung, male reproductive organs, prostate, colon, and kidney (193). The specific targeting of ERα and ERβ isoforms could be a valuable strategy for treating diseases, considering their overlapping and distinct nonoverlapping functions, as well as the tissue-specific differences in their relative abundance (187).

A plethora of research has illustrated the vital role of ER in cancer therapeutics. For instance, a number of studies have depicted that 17-β-estradiol or E2, a natural female steroid hormone, resulted in increased cell proliferation, growth, viability, and migration of various OVC cells and caused an increase in tumor growth and size *in vivo* through ER signaling pathway (7, 76, 89–93, 95, 97, 98, 102, 104, 108, 111). Moreover, many studies have clearly illustrated that ERα is often highly expressed in OVC and its natural ligand E2 has tumorigenic effects (55, 97, 104). Hung et al. reported that non-genomic ERα signaling increased the proliferation, invasion, and migration of immature ovarian teratoma cells by elevating the expression of microRNA-21 (125). Further, Parker et al. demonstrated that 24 genes specifically involved with ERs showed differential expression in various OVC cell lines, when treated with either E2 or genistein, a phytochemical with estrogenic potentials. This includes genes that regulate survival, invasion, migration, and other aspects critical to the development and progression of OVC (100). In addition, bisphenol A (BPA), a potent endocrine disrupting chemical (EDC) and an environmental estrogen mimic, was also shown to contribute significantly towards OVC progression via ER pathway by enhancing cell proliferation, adhesion, invasion, angiogenesis, and migration, as well as elevating cell growth-related genes (99, 110, 111, 117, 127). Further, di-n-butyl phthalate and hexabromocyclododecane, two EDCs were shown to induce estrogenic effects in OVC cells by inducing their proliferation (103). Therefore, a large number of synthetic ligands collectively known as selective estrogen receptor modulators (SERMs) are being employed for the therapeutic targeting of ER (187). For example, several studies suggested that treatment with tamoxifen and its derivatives profoundly reduced cell proliferation, and tumor growth, and induced apoptosis in preclinical OVC models (89, 90, 94, 98, 101, 122, 129, 194). Interestingly, a few studies explicitly conducted on ERβ indicated that E2 treatment alone or combined with ERβ overexpression resulted in reduced cell proliferation and motility by inducing the expression of p21 and fibulin-1c and downregulating the expression of MMP-2 and cyclin A2 (131, 132). Verardi et al. reported that an indole derivative interacted with ERβ and resulted in the suppression of growth of OVC cells (136). Further, another study showed that overexpression of ERβ5 promotes migration, invasion, and proliferation of OVC cells through FAK/c-Src activation establishing it as potential target for OVC treatment (134).

In addition, another study demonstrated that genistein and daidzein, two isoflavones from soy foods also exhibited anti-estrogenic effects in numerous OVC cells by inducing apoptosis, cell cycle arrest, and reducing cancer cell viability, sphere formation, invasion, and migration (96, 113). Besides, another study utilized two ERβ agonists, liquiritigenin, and S-equol, which is isolated from soy isoflavone daidzein, where both the agonists suppressed invasion and migration and induced apoptosis in preclinical models of OVC (133). Further, these compounds were reported to downregulate PI3K/Akt/Wnt signaling pathways in OVC cell lines such as SKOV3, NIH: OVCAR-3, CAOV3, and A2780 (96). Another study demonstrated that aconitine, a toxin from Aconitum plant inhibited the proliferation, motility, and colony formation along with inducing apoptosis and DNA damage of OVC cells (135). A few studies demonstrated that leptin promoted cell growth and invasion of SKOV3 cells by inducing estrogen and ERα expression. Nevertheless, treatment with selective ERα antagonist methyl-piperidine-pyrazole (MPP) substantially suppressed the growth of these cells (121, 128). Likewise, a couple of studies have reported the anti-carcinogenic effects of MPP in ERα-expressing OVC cells (106, 117). Moreover, another study showed that 3-butoxy-1,8,9-trihydroxy-6H-benzofuro [3,2-c]benzopyran-6-one (BTB), derived from wedelolactone, selectively blocked the E2-induced transactivation of ERs and hindered the growth of ER-positive OVC cells (107).

A plethora of preclinical studies provided evidence that anti-estrogen ICI 182, 780 or fulvestrant caused a significant decrease in OVC growth and metastasis by degrading ER and inducing apoptosis and cell cycle arrest (7, 94, 97, 99, 105, 108, 110, 114, 121, 122, 128). Many studies revealed that benzophenone-1 or 4-tert-octylphenol mimicked estrogenic effects by enhancing cell migration and the expression of proteins like N-cadherin, Snail, and Slug along with downregulating E-cadherin via ER-dependent manner (112, 124, 130). An interesting study reported that cadmium has strong estrogenic effects and it activated ER, which was reversed by the addition of ICI 182, 780 wherein it inhibited cell growth and both ERα and ERβ expression (115). Another study showed that suramin, an anticancer agent resulted in an unexpected increase in the growth of ER-positive OVC cells, thereby providing insights into the complex pathways through which ER modulates OVC progression (120). A number of studies have exploited the ERα specific agonistic action of 1, 3, 5-Tris (4-hydroxyphenyl)-4-propyl-1H-pyrazole (PPT) in upregulating the ERα expression, cell growth and migration in many OVC cell lines. Nonetheless, these effects could be potentially reversed by a strong antagonist of this receptor (99, 106, 123).

Another study portrayed the effects of pesticides, which are found in trace amounts in harvested fruits and vegetables on the development of OVC. This study showed that fenhexamid and cyprodinil, two potential fungicides, apparently increased cell proliferation and metastasis in BG-1 OVC cell line and increased tumor volume in mice models. However, treatment with the anti-estrogen, ICI 182, 780 suppressed cell proliferation by downregulating cathepsin D, PCNA, cyclin D, and E (109). In another study, the synergistic effect of trametinib and tamoxifen was explored in OVCAR-3 and SKOV3 cell lines previously treated with estrogen. It was found that treatment led to no significant impact on the growth of these cells. However, the treatment with trametinib or erlotinib alone showed a substantial reduction in cell growth (116). In addition, another study reported that micro RNA-26b suppressed the proliferation, invasion and migration of OVC cells by inhibiting ERα (118). Moreover, few studies have demonstrated that siRNA-mediated repression of ERα caused reduction in cell growth of various OVC cell lines and subsequent treatments with a potent ERα antagonist had enhanced anticancer effects (99, 127, 128). Interestingly, overexpression of ERβ in numerous lines of OVC cells, potentially reduced cell growth and migration and increased apoptosis (99, 131). Another study showed that the ERβ agonist, LY500307, inhibited growth, sphere formation, and self-renewal of ovarian cancer stem cells, while also inducing apoptosis and cell cycle arrest in these cells (137). Besides, another study revealed that OSU-ERb-12, an ERβ agonist suppressed OVC progression and inhibited cancer stem cell (CSC) subpopulation by preventing non-CSC to CSC conversion (119). Similarly, the treatment of OVC cell lines with the ERβ-specific antagonist 2,3-bis (4-hydroxy-phenyl)-propionitrile or DPN, also resulted in decreased growth and enhanced apoptosis (99, 106). Another study demonstrated semaphorin 4D expression, positively correlated with OVC progression, where it was upregulated by ERα which accelerated the proliferation of OVC cells, and was downregulated by ERβ which inhibited cell multiplication (126). In essence, these studies have uncovered the distinct roles of both the isoforms of ER and their agonists and antagonists that might pave the way for the therapeutic interventions of OVC.

### 2.3 Estrogen-related receptor

ERR, also known as nuclear receptor 3 group B (NR3B), has three isoforms ERRα (NR3B1), ERRβ (NR3B2), and ERRγ (NR3B3), and are located at the 11q13.1, 14q24.3, and 1q41 position respectively (https://www.genenames.org/data/gene-symbol-report/#!/hgnc_id/3471, https://www.genenames.org/data/gene-symbol-report/#!/hgnc_id/3473, https://www.genenames.org/data/gene-symbol-report/#!/hgnc_id/3474) (187). ERRα is present throughout the body except in regions like the oral mucosa, ovary, smooth muscle, spleen, and vagina. ERRβ is expressed in regions like amygdala, basal ganglia, breast, cerebellum, cerebral cortex, choroid plexus, epididymis, fallopian tube, heart muscle, hippocampal formation, hypothalamus, kidney, medulla oblongata, midbrain, pons, prostate, retina, skeletal muscle, spinal cord, spleen, and stomach, testis, thalamus, thymus, tongue, and white matter. ERRγ is found in regions like the stomach, kidney, cerebral cortex, cerebellum, nasopharynx, bronchus, lung, esophagus, colon, rectum, testis, prostate, breast, heart muscle, smooth muscle, and skeletal muscle (190). ERRs exhibit a relatively similar sequence to that of ERs, particularly in the DBD regions, with a sequence identity of over 60% between ERRα and ERα (195). ERRs have been found to exert differential effects on estrogen signaling, and studies examining ERR expression in ovarian tumors have identified ERRα and ERRγ as potential prognostic markers for these cancers (196, 197). Moreover, studies have also reported that ERRα, ERRβ, and ERRγ are upregulated in OVC tissues compared to normal ovarian tissues (63, 64). An interesting study revealed that the overexpression of ERRα elevated the levels of Snail and promoted EMT whereas specific inhibition of ERRα led to the suppression of Snail, upregulation of E-cadherin expression, and reduction in stem cell properties. It was found that the miR-200 family has a role in the post-transcriptional control of Snail mediated by ERRα. Inhibition of miR-200a/b was found to counteract the downregulation of Snail caused by ERRα depletion *in vitro*. Further, the silencing of ERRα *in vivo* led to a substantial decrease in tumor burden, ascites formation, and metastatic peritoneal nodules (138). In addition, Wang et al. reported that cordycepin, an antitumor compound inhibited ERRα (a co-TF associated with mitochondrial fusion), EMT, metastasis and migration of OVC cells by repressing their mitochondrial activity (139). However, more studies in the future could lead to a better understanding of ERR action in OVC.

### 2.4 Glucocorticoid receptor

GR is a glucocorticoid-activated receptor protein that belongs to the superfamily 3 of the NRs. It is encoded by a single gene, NR3C1 and is positioned at 5q31–32, and contains a highly conserved sequence (187). GRs are expressed throughout the human body and can regulate the transcription of various genes involved in development, metabolism, and inflammation by binding to various glucocorticoid response elements (GRE). Their effects on cancer cells vary depending on the cell type, making their use in cancer therapy context-dependent (198). However, in OVC, this receptor shows upregulated expression and has tumorigenic potentials (58, 144).

Several lines of evidence implicated that a synthetic glucocorticoid, dexamethasone (DEX) plays a vital role in regulating cell proliferation and survival in different OVC cell lines. For instance, Yin and colleagues revealed that DEX upregulated fibronectin and MUC1 resulting in enhanced cell adhesion and survival by upregulating PI3K/Akt pathway and survival genes in SKOV3 and HO-8910 cell lines (144). Another study reported that the treatment of OVC cell lines with DEX resulted in suppression of paclitaxel-mediated apoptosis. DEX upregulated two important pro-survival genes, serum and glucocorticoid-regulated kinase 1 (SGK1), and map kinase phosphatase 1 (MKP1)/dual specificity phosphatase 1 (DUSP1) (142). Further, these effects were evident in human trials, where pharmacological doses of DEX were administered to patients undergoing chemotherapy (142). In essence, these results suggest that activation of the GR may lead to an increase in anti-apoptotic gene expression in cancer patients, thus hindering chemotherapy-induced apoptosis (142). Accordingly, similar results were observed when OVC cells were treated with DEX in combination with carboplatin and gemcitabine. Here, DEX upregulated SGK1 and MKP1/DUSP1 and significantly suppressed carboplatin or gemcitabine-induced cell death. However, the treatment with GR antagonists, mifepristone, or CORT125134 partially abrogated this effect. Further, a significant decrease in tumor volume was observed when OVC xenografts were treated with carboplatin or gemcitabine along with mifepristone (143). These results suggested that antagonists of GR enhanced the sensitivity of GR-positive OVC cells to chemotherapy-induced cell death by impeding the GR-mediated cellular survival pathways (143). Furthermore, preclinical and clinical studies revealed that the selective GR modulation via relacorilant (a selective glucocorticoid receptor modulator), when used in combination with nab-paclitaxel, may result in enhanced efficacy of chemotherapy (146). Another study demonstrated that DEX inhibited the proliferation of 3AO cells in both time and concentration-dependent manner. Additionally, treatment with DEX resulted in an increase in alkaline phosphatase (ALP) activity and a decrease in the expression of the CA125 tumor marker in 3AO cells. These findings suggested that glucocorticoids are critical regulators of 3AO cell proliferation and differentiation (140). Moreover, an *in vitro* study demonstrated that 3AO cells express functional GR that can be downregulated by DEX at both protein and mRNA levels (141). ORIC-101 (structure-based modified mifepristone), is a highly potent steroidal GR antagonist with reduced AR agonistic activity, making it suitable for use in AR-positive tumors. It has also shown an improved inhibition profile for CYP2C8 and CYP2C9, reducing the potential for drug-drug interactions. Unlike mifepristone, ORIC-101 can be co-administered with chemotherapeutic agents metabolized by CYP2C8, such as paclitaxel. Furthermore, ORIC-101 exhibited antitumor activity *in vivo* by enhancing the chemotherapy response in the GR-positive OVCAR-5 OVC xenograft model. Moreover, the safety and therapeutic potential of ORIC-101 is currently being evaluated in clinical studies (145). Taken together, these findings suggest a crucial role of GR in ovarian tumorigenesis, and effective interventions of GR at clinical levels could be a valuable therapeutic option targeting OVC.

### 2.5 NR1D1

Nuclear receptor subfamily 1 group D member 1 (NR1D1), also known as REV-ERBα, is a member of the nuclear hormone receptor family that plays a critical role in the regulation of various physiological processes, including metabolism, inflammation, and circadian rhythm (199, 200). Several studies over the years have uncovered the role of NR1D1 in the pathophysiology of cancer (201, 202). Wang et al. demonstrated that overexpression of NR1D1 suppressed proliferation and induced apoptosis of OVCAR-3 cells. Further, overexpression of NR1D1 remarkably downregulated JAK/STAT3 pathway by upregulating suppressor of cytokine signaling (SOCS) 3 in both OVC cell lines and xenografts (147). However, more studies are warranted to validate the tumor suppressor function of this receptor in OVC.

### 2.6 Nur77/TR3

Nur77, also known as NR4A1, NGFI-B, or TR3, is a member of the NR superfamily that has a role in the regulation of both the survival and death of cancerous cells (187, 203, 204). It is located at the 12q13.13 position of the chromosome (189). Nur77 is expressed throughout the body except in regions like the adipose tissue, bone marrow, liver, parathyroid gland, prostate, and spleen (190). It has been found that Nur77 signaling is dysregulated in numerous types of cancer and serves as a crucial target for cancer therapy (205). For instance, vitamin K2 treatment of PA-1 OVC cell line resulted in increased Nur77 expression in both the mitochondria and nuclei potentially implicating the induction of apoptosis mediated by vitamin K2, suggesting that this vitamin may possess therapeutic potential for the treatment of OVC (148). Another *in vitro* study showed that Nur77 is a crucial regulator of apoptosis and a key mediator of the response to cytotoxic chemotherapy, such as cisplatin, in OVC. Nevertheless, this study also suggested that upregulating Nur77 expression and promoting its nuclear export could represent a rational therapeutic strategy for counteracting cisplatin resistance in OVC (149). Another study reported that the expression of Nur77 is higher in ovarian tissue samples than in other tissues and the overexpression of this orphan NR is correlated with worse PFS. Besides, Nur77 expression showed heterogeneity among different high grade serous ovarian carcinoma (HGSOC) cell lines and samples and showed localization in cytoplasm and nucleus (206). However, further research is crucial to understand the functional role of Nur77 in modulating ovarian tumorigenesis.

### 2.7 Progesterone receptor

The PR or NR3C2 is a hormone-regulated TF located on chromosome 11 at q22 position and employs two different promoters to give rise to isoforms, PR-A and PR-B. The well-documented ligand of PR is progesterone (4-pregnene-3,20-dione) or P4 and both the ligand and its receptor play an inevitable role in the regulation of reproductive genes. Upon binding of ligand, PR dimerizes and enters the nucleus where it binds to progesterone receptor DNA-response element (PRE) and regulates the transcription of numerous genes (187, 198). Importantly, both the isoforms of PR are shown to be highly expressed in OVC issues than in normal ovaries and hence ligands/agents that target PR could act as potential therapeutic agents against this cancer (68).

Several lines of evidence have unequivocally proved that P4 binding to PR and the subsequent upregulation of this receptor have protective effects on cancer development (68, 91). Various studies have revealed that estrogen and its analogs greatly induce the expression of PR and increase the growth and proliferation of OVC cells (68, 91, 165). Besides, this hormone also induces tumor growth and volume in xenograft models. However, when OVC cells were treated with estrogen alone or combined with P4, cell proliferation, survival, and tumor growth were reduced significantly (68, 91). Another study demonstrated that tamoxifen and megestrol acetate (a progestin medication) substantially reduced tumor volume in PE04 cell xenografts (93). Another study reported that primary cell cultures derived from patients with EOC revealed that the isolated cells expressed ER and PR, with the ER positive/PR positive combination being the most prevalent. After a 72 hour culture period, both ER and PR expression levels declined. The survival rates of cells cultured in P4 appeared inversely correlated with PR expression, while reductions in 17-β-estradiol and testosterone levels in the cultures were associated with decreased cell survival. These findings suggest a significant impact of sex steroids on PR expression and the survival of ovarian epithelial tumor cells (162). Takahashi et al. demonstrated that treatment with cAMP activated the PR-B, leading to cellular senescence and suppression of tumorigenicity in SKOV (p16 and p53 null) OVC cells (163). Another study revealed that P4 safeguards OVC cells from cisplatin -induced apoptosis by activating the PI3K/Akt survival pathway and modulating the expression profiles of PGR and 1/2. Consequently, combining PI3K/Akt signaling inhibitors or a PGRMC antagonist with platinum-based chemotherapy could improve treatment outcomes and prognosis in OVC patients (164). Lima et al. showed that P4 enhanced the expression levels of a disintegrin and metalloproteinase with thrombospondin motifs (ADAMTS) in lysates from ES2 cells, and in lysates and conditioned media from NIH-OVCAR-3 cells, suggesting P4 acts via the PR to regulate ADAMTS levels (1 and 4) in OVC cells (166). Another study reported that progestins and vitamin D exhibit a synergistic effect in reducing cell viability and inducing apoptosis in OVC cells. Progestins also inhibit calcitriol (CAL)-induced CYP24A1 expression through PR-dependent mechanisms, thereby prolonging CAL activity. This combination therapy of progestins and vitamin D warrants further investigation as a potential strategy to inhibit ovarian carcinogenesis (167). Additionally, another study highlighted the critical role of P4 and the presence of PRs in reducing disease progression in endometrioid ovarian carcinoma (168). Further, HGSOC tumors exhibiting weak PR-B expression were linked to increased platinum resistance and poor survival outcomes. In preclinical settings, P4 and PR-B expression enhanced the sensitivity of HGSC cells to cisplatin by promoting cisplatin-induced apoptosis. Furthermore, preclinical evaluations demonstrated that P4 treatment could re-sensitize platinum-resistant HGSC cells to cisplatin, suggesting this approach could be clinically valuable in enhancing the effectiveness of cisplatin therapy in the treatment and management of OVC (50). Although the role of PR in OVC has been elucidated, further research is required to identify agents that could modulate the PR expression as a viable strategy in the treatment of OVC.

### 2.8 Peroxisome-proliferator-activator receptor

PPAR, also known as nuclear receptor 1, group 3 has three isoforms PPAR α (NR1C1), PPAR β (NR1C2), and PPARγ (NR1C3), and regulates energy metabolism (187, 207). These isoforms are located at 22q13.31, 6p21.31, and 3p25.2 positions (https://www.genenames.org/data/gene-symbol-report/#!/hgnc_id/9232; https://www.genenames.org/data/gene-symbol-report/#!/hgnc_id/9235; https://www.genenames.org/data/gene-symbol-report/#!/hgnc_id/9236). PPARα is expressed throughout the body. PPARβ expression is found in regions like the liver, prostate, heart, and skeletal muscles. PPARγ is present in the regions like appendix, cervix, colon, epididymis, esophagus, oral mucosa, placenta, rectum, stomach, tonsil, urinary bladder, adrenal gland, duodenum, gallbladder, hippocampus, lung, salivary gland, skin, small intestine, thyroid gland, vagina, adipose tissue, bone marrow, breast, bronchus, caudate, cerebellum cerebral cortex, kidney, skeletal muscle, spleen, and testis (190). In addition, PPARs are significantly expressed in the immune cells, which displayed their crucial involvement in immune cell differentiation and fate determination (207). PPARα and PPARδ play a pivotal role in regulating genes associated with substrate delivery, oxidative phosphorylation, and the maintenance of energy homeostasis (207). On the other hand, PPARγ primarily governs lipogenesis and lipid synthesis, with the highest expression levels observed in white adipose tissue (207). PPARs also differ in ligand specificity, and response to agonists and antagonists (208). PPARs have emerged as promising therapeutic targets for a wide range of diseases, such as metabolic, autoimmune, and cancers, including OVC (208–213).

Studies have reported the upregulation of PPAR in OVC compared to adjacent normal tissues (66, 67). An intriguing study reported that the treatment of oroxylin A (OA), a phytochemical, upregulated the expression of PPARγ and inhibited both the migratory capacity and viability of SKOV3 cells. Further, OA induced apoptotic cell death and restored the expression of the PGRMC1/2 family in OVC cells (159). Leng and colleagues demonstrated that low-level exposure to mono(2-ethylhexyl) phthalate (MEHP), a major toxicant from plastics activated the PI3K/Akt/NF-κB pathway in a PPARα-dependent manner, promoting OVC progression in both *in vitro* and *in vivo* (160). Several studies have reported that the treatment of PPAR modulators, such as ciglitazone, pioglitazone, troglitazone, clofibric acid, GW9662, GW6471 and rosiglitazone alone or in combination with other compounds like BPA, clioquinol, and DHA, resulted in multiple anticancer activities such as induction of apoptosis, necrosis and suppression of cell growth, viability, colony formation and glucose intake. These studies also showed that treatment of OVC cells with these compounds reduced tumor growth, inhibition of MMP-9, and COX-2 expression in pre-clinical settings in a PPAR-dependent manner (51, 67, 151–156). In addition, Hoffmann and team reported that BPA promoted the expression of apelin, which is an endogenous adipokine involved in the proliferation and migration of many cancers, in a PPARγ-dependent manner (158). However, a different study conducted by this team reported that BPA and its derivatives suppressed the expression of chemerin, another adipokine, thereby inducing the proliferation of OVC cells (51). Further, another study reported that the treatment of telmisartan, an angiotensin receptor blocker, resulted in the induction of apoptosis, activation of caspase-3, upregulation of PPARγ expression, and inhibition of cell growth and MMP-9 expression in HEY cells (157). Collectively, these studies suggest that targeting PPAR could result in several antitumorigenic effects leading to better management and treatment of OVC.

### 2.9 Retinoic acid receptor

RAR belongs to the nuclear receptor subfamily 1 group B of the NR superfamily, which binds to ligands such as RA and its isomers. RAR has three isoforms RARα, RARβ, and RARγ located on chromosomes, 17q21.2, 3p24.2, and 12q13.13, respectively (189, 214). RARs and RXRs are both regulated by their natural ligand retinoids, which upon binding regulate the transcription of several important genes. The dysregulation of the RA signaling pathway is suggested to be the underlying cause of a broad range of malignancies, including OVC, leukemia, skin cancer, head and neck cancer, lung cancer, breast cancer, prostate cancer, renal cell carcinoma, pancreatic cancer, liver cancer, glioblastoma, and neuroblastoma (214). RARα and RARγ are expressed throughout the body, but the expression of RARβ is constricted to regions except parathyroid gland, duodenum, liver, kidney, prostate, ovary, heart muscle, adipose tissue, lymph node, tonsil, and bone marrow (190). Treatment of OVC cell lines either alone or in combination with retinoids, and their derivatives, forskolin, 8-Cl-cAMP, 6-[-(1-Admantyl)-4-hydroxyphenyl]-2-naphthalene carboxylic acid (AHPN/CD437), N-(4-hydroxyphenyl) retinamide (4HPR or fenretinide), N-(4-methoxyphenyl) retinamide (4MPR), PD153035, and 2-(6-carboxy-2-naphthalenyl)-2-(5,6,7,8-tetrahydro-5,5,8,8-tetramethyl-2-naphthalenyl)-1,3-dithione (MM11253) inhibited different hallmarks of cancer such as growth, proliferation, colony formation, and apoptosis (56, 169–173). For instance, OVC cells when treated with the combination of RA and 8-Cl-cAMP resulted in increased RARβ expression followed by a reduction in colony formation ability by initiating fragmentation of nucleosomes resulting in apoptosis, as evidenced by an increase in the expression of cleaved caspase-3 and PARP (170). Another study demonstrated that overexpression of RARα and RXRα resulted in significant growth inhibition in both SKOV3 and CAOV3 cells when treated with RA. This study suggests that RAR/RXR profiles could be valuable in determining the potential therapeutic benefits of RA or receptor-specific retinoid derivatives in treating OVC (56). Another study showed that the treatment of OVCAR-3 cells with EGFR inhibitor PD153035 resulted in cellular inhibition and the induction of RARβ expression through the demethylation of its promoter sequences (174). In addition, siRNA-mediated knockdown of RARγ led to decreased cell proliferation and colony number in OVC cells. Further, the knockdown of RARγ in A2780 xenografts resulted in decreased tumor weight and volume along with reduced expression of RARγ, Ki-67, and PCNA (49). Another study showed that RARβ is involved in reducing the proliferation of OVC cells and increasing the sensitivity of these cells to a synthetic retinoid derivative, N-(4-hydroxyphenyl) retinamide (4HPR or fenretinide), suggesting RARs might have a tumor-suppressing effect in ovarian tumorigenesis (172). Taken together, these results suggest that further research is needed to better understand the role of RARs in OVC, which could provide a more holistic picture of the mechanistic action of this receptor in ovarian tumor biology.

### 2.10 Retinoid X receptor

In 1990, Mangelsdorf et al. discovered RXRs as orphan receptors, initially identified without any known ligands (215). However, the high sequence similarity between RXRs and RARs, along with their capacity to activate various genes in the presence of all-trans RA, highlighted their close association with RARs (216). Subsequently, 9-cis-RA was identified as their natural ligand (22, 215). RXRs exist in three distinct isoforms in mammals: RXRα, RXRβ, and RXRγ, also known as NR2B1, NR2B2, and NR2B3. These isoforms are encoded by separate genes located on human chromosomes 9q34.2, 6p21.32, and 1q23.3, respectively (215). Several studies have demonstrated the role of RXRs in OVC development. For instance, Holmes et al. reported that there is a higher apoptotic index in OVC cells overexpressing RXRα than in parental cells (171). In addition, another study showed that resveratrol induced apoptosis in carboplatin-resistant OVC cells by elevating the levels of RXRα and downregulating sirtunin 1 expression (175). Further, another study revealed the responsiveness of OVC cells to all-trans RA treatment depends significantly on the expression levels and activity of RARs and RXRs, particularly RARα and RXRα. This study found that modulating the expression of these receptors in resistant cell lines can restore sensitivity to RA, leading to effective therapeutic strategies for this cancer (56).

### 2.11 Vitamin D receptor

VDR belongs to the NR subfamily 1 group I of the nuclear receptor superfamily located on 12q13.11 (189). Upon activation with endogenous ligands, VDR modulates the expression of its target genes by heterodimerizing with RXR isoforms that translocate to the nucleus and bind to the vitamin D response elements (217).

VDR regulates different cellular processes such as cell cycle progression, proliferation, growth, survival, apoptosis, etc., upon activation with its ligands alone or in combination, especially with 1α,25-dihydroxy vitamin D3, MT19c, calcitriol, progesterone, cisplatin, paclitaxel, carboplatin, and synthetic vitamin D analogs (48, 183, 185). For instance, treatment with 1, 25VD suppressed the growth of OVCAR-3 cells and induced cell cycle arrest by inhibiting Cdc2 kinase and cyclin B1 levels. In addition, GADD45 was identified as an immediate early response gene that mediated the inhibitory effects of 1, 25VD on cell growth and cell cycle progression (176). In addition, it has been reported that vitamin D3 inhibits the growth and migration of the A2780 cells by phosphorylating MAPK and Akt proteins. Besides, it also inhibited metastatic potential and induced apoptosis in murine teratocarcinoma cells. However, this study also showed that vitamin D3 stimulated the growth of normal embryonic stem cells. Therefore, this study suggests that vitamin D3 may inhibit the proliferation of malignant cells while potentially protecting normal stem cells crucial for development and tissue regeneration (180). Another study reported that the treatment of ES2 OVC cells with a combination of calcitriol and progesterone, induced VDR expression and suppressed cell proliferation along with abating the expression of TGF-β, SMAD signaling proteins, and CYP24A1 (48). An intriguing study showed that treatment of OVC cells with a combination of vitamin D and TGF-β1 to OVC cells resulted in the reversal of TGF-β1 induced EMT by restoring the E-cadherin coupled with decreasing α-SMA, N-cadherin, slug, MMP-2, and MMP-9 expression (186). Another study showed that the treatment of SKOV3 cells with VDR antagonist, MT19c exhibited decreased cell proliferation, increased cell cycle arrest, and induced caspase-dependent apoptosis. Further, it reduced the IRS-1/2 pathway and its downstream target genes by MAPK/JNK activation (177). In addition, another study showed that MT19c specifically targets the metabolic pathways of cancer cells, particularly by inhibiting fatty acid synthase (FASN) functions and disrupting de novo lipogenesis, which is a characteristic feature of cancer cell metabolism in both in vitro and in vivo (179). Another study demonstrated that treatment with progesterone and calcitriol led to the inhibition of IκBα phosphorylation, suppression of NF-κB activation, and reduced expression of NF-κB-regulated genes that promote metastasis. These findings suggest that progesterone and calcitriol could be effective in managing ovarian tumors (178). Ji et al. demonstrated that 1α,25-dihydroxyvitamin D3 (1α,25(OH)2D3) restricts the stem cell-like properties of OVC cells by upregulating the expression of VDR, promoting the cytoplasmic β-catenin expression, and suppressing the CD44 expression (181). Another study demonstrated that 1α,25(OH)2D3 enhances radiosensitivity in a VDR-dependent manner and activates the NADPH oxidase- ROS-apoptosis pathway. This suggests that combining 1α,25(OH)2D3 with radiation therapy could enhance radiosensitivity in ovarian cells, potentially offering a novel therapeutic strategy for this disease (182). Further, it was reported that the combined treatment of calcitriol and cisplatin in SKOV3 OVC cells resulted in enhanced antiproliferative, apoptotic, and anti-angiogenic effects compared to cisplatin alone (184). Collectively, these studies indicate that targeting the VDR with its modulators induces various anticancer effects, including apoptosis, cell cycle arrest, inhibition of cell growth, and reduction of tumor growth, suggesting it might positively impact the treatment and management of OVC.

## 3 Clinical trials of NR-targeted drugs in OVC

Multiple clinical studies have been carried out in OVC patients for developing potential NR-based anticancer drugs. The clinical trials employing agonists/antagonists of NRs have been summarized in **Table 3** (116, 142, 146, 218–232). For instance, the treatment of anti-estrogen fulvestrant to ER-positive recurrent ovarian cancer patients resulted in enhanced progression-free survival (PFS) of these patients (219). Another study revealed that the administration of everolimus and letrozole in combination resulted in 47% 12-week PFS in nine out of nineteen ER-positive HGSOC patients enrolled (220). Further, several studies engaged the treatment of recurrent/metastatic OVC patients with the aromatase inhibitor anastrozole, which resulted in increased PFS and tumoricidal activity and reduced inhibin levels, pain, and fatigue (222–225, 228). Furthermore, anastrozole and everolimus treatment resulted in complete or partial response in a significant percentage of patients with aberrations in multiple signaling pathways (232). Many clinical trials have been conducted where an aromatase inhibitor, letrozole alone or in combination with ribociclib was administered to OVC patients. As a result, the overall clinical benefit rate (CBR) and survival of patients were improved (226, 227, 229, 230). Another study reported that treating stage III or IV EOC patients with tamoxifen resulted in elevated ER levels and achieved 10% complete response and 8% partial response (221). In another study, a 28-year-old low grade OVC patient, when treated with trametinib and tamoxifen combination, increased tumor mass and cancer antigen 125 (CA125) levels. However, combinatorial treatment of trametinib and letrozole resulted in reduced tumor mass and CA125 (116). Nonetheless, another study revealed that ERβ expression led to poor survival in patients treated with carboplatin/docetaxel with or without celecoxib (231). Another study showed that treatment of OVC patients with DEX led to the increased expression of prosurvival genes such as SGK1 and MKP1/DUSP1, suggesting that high doses of glucocorticoids might reduce chemotherapy effectiveness by enhancing anti-apoptotic gene expression (142). Further, Munster et al. evaluated the combinatorial treatment of GR modulator relacorilant and paclitaxel in OVC patients which resulted in an increased response to paclitaxel with minimal toxicity (146). Cancer of the ovary abiraterone or CORAL represents the first ever AR-targeted phase II clinical trial initiated in AR-positive EOC patients. Even though the response to the abiraterone was minimal, a subset of the patients showed enhanced CBR (218). Therefore, an extensive evaluation of clinical studies and their outcomes is crucial and unavoidable for the development of effective NR-targeted drugs for the treatment of OVC. Besides, more studies are needed to understand the potential and efficacy of these drugs in clinical settings.

**Table 3:** Clinical studies of nuclear receptors agonists/antagonists in ovarian cancer patients.

Agent	Trial Identifier/ PMID	Patient population	Results	Current status	References
Androgen Receptor (AR)					
Abiraterone (Phase II)	ISRCTN63407050	EOC patients	CBR rate-26% ORR-2%	Completed	(218)
Estrogen Receptor (ER)					
Fulvestrant (Phase II)	PMID: 19239974	ER^+VE^ EOC patients	↑PFS CR-4%, Partial response-4%, SD-35% (modified-Rustin criteria) SD-50% (modified-RECIST criteria)	Completed	(219)
Everolimus+ Letrozole (Phase II)	NCT02283658	High grade ovarian carcinoma patients	PFS at 12 weeks-47%	Completed	(220)
Tamoxifen	PMID: 2070324	Stage III or IV EOC patients	↑CR-10%, Partial response-8%	Completed	(221)
Anastrozole (Phase II)	PMID: 31227223	Recurrent/metastatic low grade and serous borderline OVC patients	↑CBR SD-50% (18/36) ↓Pain, fatigue	–	(222)
Anastrozole (Phase II)	PMID: 31328463	ER^+VE^/PR^+VE^ Post-menopausal EOC patients	↑Antitumor activity CBR at 3 months-34.6% No significant effect on ER levels	–	(223)
Anastrozole (Phase II)	PMID: 34412908	Metastatic ovarian GCT patients	↑PFS ↓Inhibin levels	–	(224)
Anastrozole (Phase II)	ACTRN12610000796088	ER^+VE^/PR^+VE^ post-menopausal women with recurrent ovarian cancer	↑PFS, CBR-27% (13/49) ↓ER	–	(225)
Ribociclib+ Letrozole	NCT02657928	Relapsed ER-positive ovarian cancer patients	↑Positive survival effects PFS at 12 weeks-50%	Completed	(226)
Letrozole (Phase II)	PMID: 18457865	ER+ platinum- and taxane-resistant high-grade ovarian cancer patients	↑CBR	–	(227)
Anastrozole (Phase II)	PMID: 14675683	Ovarian carcinoma patients	↑tumoricidal activity, PFS	–	(228)
Letrozole (Phase II)	PMID: 17575226	ER^+VE^/PR^+VE^ ovarian cancer patients	↑Survival ↓HER2 expression, CA-125	–	(229)
Letrozole	PMID: 12114425	Ovarian cancer patients	↑ER, EGFR, survival ↓ERβ2	–	(230)
Carboplatin/docetaxel ± celecoxib (Phase II)	PMID: 26115976	Epithelial ovarian carcinoma patients	↑ERβ ↓PFS, OS	–	(231)
Anastrozole+ Everolimus	NCT01197170	ER+ Ovarian cancer patients	↑CCNE1, IRS2, MCL1, CCND1, FGFR1 and MYC Alterations in PI3K/Akt/mTOR pathway, rearrangement of PRKDC	Completed	(232)
Trametinib+ Tamoxifen	PMID: 33528846	28-year-old LGSOC patient	↑Tumor mass, CA125	Completed	(116)
Trametinib+ Letrozole	PMID: 33528846	28-year-old LGSOC patient	↓Tumor mass, CA125	Completed	(116)
Glucocorticoid receptor (GR)					
Dexamethasone	PMID: 19383827	Ovarian and primary peritoneal cancer patients	↑GR, SGK1, MKP1/DUSP1	Completed	(142)
Nab-paclitaxel+ Relacorilant (Phase II)	PMID: 35583817	Ovarian cancer patients with advanced or metastatic solid tumors	↑PFS, Clinical benefit Durable disease control-33% Longer duration of benefit than taxane-28%	Completed	(146)
Progesterone Receptor (PR)					
Anastrozole+ Everolimus (Phase I)	NCT01197170	PR^+VE^ ovarian cancer patients	CR-2/10 patients (20%) ↑CCNE1, IRS2, MCL1, CCND1, FGFR1 and MYC Alterations in PI3K/Akt/mTOR pathway, rearrangement of PRKDC	Completed	(232)

α-SMA- α-smooth muscle actin; 4HPR-N-(4-hydroxyphenyl) retinamide; ADAMTS- A disintegrin and metalloproteinase with thrombospondin motifs; AF1-Activation function 1; AF2-Activation function 1; AHPN/CD437–6-[-(1-Admantyl)-4-hydroxyphenyl]-2-naphthalenecarboxylic acid; ALP- Alkaline phosphatase; AR-Androgen receptor; BPA- Bisphenol A; BTB- 3-butoxy-1,8,9-trihydroxy-6H-benzofuro[3,2-c] benzopyran-6-one; CAL- Calcitriol; CA-125-Cancer antigen 125; CBR- Clinical beneficial rate; CGZ- Ciglitazone, Co-OE-Co-overexpression; CR-Complete response; CSC- Cancer stem cells; CSPC-Cancer stem/progenitor cells; DBD- DNA-binding domain; DHT- dihydrotestosterone; DEX- Dexamethasone; DPN- 2,3-bis(4-hydroxy-phenyl)-propionitrile; E2- 17-β‐estradiol; EDC-Endocrine disrupting chemical; EMT-Epithelial-mesenchymal transition; EOC-Epithelial ovarian carcinoma; ER-Estrogen receptor; FAK-Focal adhesion kinase; FDA-Food and Drug Administration; FKBP5-FK506-binding protein 5; GCT-granulosa cell tumors; GR-Glucocorticoid receptor; GRE- Glucocorticoid response elements; hFTE cells-Human fallopian tube epithelial cells; HGSOC- High grade serous ovarian carcinoma; IMAC-1- intercellular cell adhesion molecule-1; IRF1- interferon regulatory factor 1; LBD-Ligand-binding domain; LGOCs- low-grade ovarian cancers; MEHP-mono (2-ethylhexyl) phthalate; MKP1/DUSP1-map kinase phosphatase 1/dual specificity phosphatase 1; MMP-Matrix metalloproteinases; NR- Nuclear receptor; MPA-Medroxyprogesterone acetate; MPP- Methyl-piperidino-pyrazole; OCSCs-Ovarian cancer stem cells; OCSS-Ovarian cancer specific survival; OE-Overexpression; ORR-Overall response rate; OS-Overall survival; OVC-Ovarian cancer; OVTC- ovarian teratocarcinoma; PFS- Progression-free survival; PARP- Poly (ADP)-ribose polymerase; PGZ- Pioglitazone; PPAR-Peroxisome-proliferator-activator receptor; PPT- 1,3,5-Tris(4-hydroxyphenyl)-4-propyl-1H-pyrazole; PR-Progesterone receptor; PRE- Progesterone receptor DNA-response element; RA-Retinoic acid; RAR-Retinoic acid receptor; RXR-Retinoid X receptor; SBOTs- serous borderline ovarian tumors; SD-Stable disease; SERMs-Selective estrogen receptor modulators; SGK1-serum and glucocorticoid-regulated kinase 1; SGTA-Small glutamine-rich tetratricopeptide repeat-containing protein alpha; TBBPA-Tetrabromobisphenol A; TCBPA-Tetrachlorobisphenol A; TF-Transcription factor; TFF1-Tertoil factor 1; TGZ-troglitazone; TLR4- toll-like receptor 4; TTNN-5’,6’,7’,8’-tetrahydro-5’,5’,8’,8’-tetramethyl- [2,2’-binaphthalene]-6-carboxylic acid; TTNPB-(E)-4-[2-(5,6,7,8-tetrahydro-5,5,8,8,- tetramethyl-2-naphthalenyl)- 1-propenyl] benzoic acid; VDR-Vitamin D receptor.↓, Downregulation/inhibition; ↑, Upregulation/activation.

## 4 Conclusion

OVC, being one of the most dreadful diseases, kills more than two lakhs women around the globe annually. The current therapeutic regimens have led to increased drug resistance, debilitating side effects, and toxicity in patients. However, understanding the causative factors and disease etiology could help in identifying targets for the development of treatment regimens against OVC. NRs are a family of TFs that are activated by various small molecule ligands, such as hormones, and can modulate the expression of genes involved in development, metabolism, and inflammation. This comprehensive review has elaborated the role of NRs as promising therapeutic targets in OVC, where they regulate cell signaling pathways and transcription of genes associated with cancer cell survival, proliferation, EMT, invasion, angiogenesis, and migration. Further, studies involving agonists and antagonists of NRs investigated in preclinical and clinical settings, showing promising results in reducing tumor growth and promoting apoptosis have also been briefly described. For instance, anastrozole, letrozole, ribociclib, tamoxifen, bicalutamide, enzalutamide, fulvestrant, etc., have been successfully established as NR-targeted therapeutic interventions for OVC.

It is worth noting that NR-targeted therapeutic interventions for OVC present several strengths, limitations, and challenges. A notable strength of these therapies is their capacity to precisely target and modulate the activity of NRs, which are integral to the regulation of cell proliferation, apoptosis, and hormone signaling in OVC. For instance, several aforementioned studies have shown that targeting ERs can be particularly effective in ER-positive subtypes of OVC, potentially reducing tumor growth and enhancing patient outcomes. Additionally, the use of NR agonists and antagonists can increase the sensitivity of OVC cells to various chemotherapeutic agents, offering a strategic advantage in treatment protocols. It is worth noting that letrozole (Femara^®^), anastrozole (Arimidex^®^), and fulvestrant (Faslodex^®^) are FDA-approved drugs widely used for hormone receptor-positive metastatic breast cancers (233–235). However, they may be used off-label or in clinical trials for OVC treatment, especially when there is evidence of hormonal involvement in the tumor’s growth dynamics. Nevertheless, these therapeutic strategies also encounter significant limitations. The heterogeneity of OVC suggests that responses to these therapies can vary widely among patients, complicating the standardization of treatment approaches. Besides, the modulation of hormonal pathways can lead to potential side effects that affect a broad spectrum of bodily functions, potentially causing considerable discomfort or adverse health effects in patients. It is also noteworthy that most NR-targeted therapies demonstrate effectiveness primarily in hormone-positive types of OVC.

Therefore, the identification of NR ligands and the development of selective, safe, and efficacious modulators may provide new therapeutic options for OVC. Further research into the molecular and cellular mechanisms underlying the role of NRs in OVC is needed to develop effective strategies for early detection and targeted therapies. Conclusively, as NRs have already revolutionized cancer treatment strategies in the past, they will likely continue as a major source of novel approaches to OVC therapeutics in the future.

## Author contributions

AS: Conceptualization, Data curation, Investigation, Methodology, Writing – original draft. BB: Conceptualization, Methodology, Writing – original draft. MM: Conceptualization, Investigation, Writing – review & editing. MSA: Conceptualization, Investigation, Writing – review & editing. MA: Conceptualization, Investigation, Writing – review & editing. MS: Conceptualization, Investigation, Writing – review & editing. GS: Conceptualization, Writing – review & editing. ZM: Conceptualization, Funding acquisition, Investigation, Resources, Writing – review & editing. AK: Conceptualization, Funding acquisition, Investigation, Resources, Writing – review & editing.
